# Supplementary material for: A Bayesian inference method to estimate transmission trees with multiple introductions; applied to SARS-CoV-2 in Dutch mink farms
Source: PLoS Comput Biol. 2023 Nov 27;19(11):e1010928. doi: 10.1371/journal.pcbi.1010928 (PMC10703282; doi:10.1371/journal.pcbi.1010928)
Supplement: S1 Results — (PDF) [file pcbi.1010928.s001.pdf]

Supplementary Information

Table A: Comparison between MCMC and MC^3. Differences between median posterior log-likelihood and the log-likelihood of the simulated outbreak. Results are the means from analyses of 25 outbreaks for each setting, of the 10,001st to 35,000th MCMC cycle of each outbreak analysis.

Simulated numbers of introductions	Method	Log-likelihood
1	MCMC random	68.3
	p(MC^3) random	70.1
	MCMC NJ	67.9
	p(MC^3) NJ	70.1
5	MCMC random	27.3
	p(MC^3) random	27.0
	MCMC NJ	27.5
	p(MC^3) NJ	27.0
10	MCMC random	**9.78**
	p(MC^3) random	20.0
	MCMC NJ	17.0
	p(MC^3) NJ	20.2
15	MCMC random	**-2.04**
	p(MC^3) random	14.5
	MCMC NJ	9.49
	p(MC^3) NJ	14.5

Table B: Inferring multiple introductions with varying prior information: no information, informative priors, and fixed parameters. 25 outbreaks of size 20 are simulated with 5 introductions for each set of priors. The results of the model parameters are mean differences between mean estimates and the simulated value.

	**No information** ^{c}	**Informative priors** ^{b}	**Fixed parameters** ^{a}
**Mean difference** between estimations and simulated value			
Introductions	0.16	0.12	0.41
μ	$3.28 \cdot 10^{-5}$	$4.59 \cdot 10^{-6}$	0
m_G	0.22	0.05	0
m_S	0.40	0.02	0
r	0.08	0.10	0
r_{history}	15.1	4.95	0
**Tree inference**			
True infectors with highest support	15/20	15/20	15.7/20
True infectors in 95% CI	19.8/20	20/20	19.5/20

^{a} $\mu_G = 1$, $\sigma_G = \infty$, $\mu_S = 1$, $\sigma_S = \infty$, $\mu_\mu = 0$, $\sigma_\mu = 100$

^{b} $\mu_G = 1$, $\sigma_G = 0.1$, $\mu_S = 1$, $\sigma_S = 0.1$, $\mu_\mu = 10^{-4}$, $\sigma_\mu = 5 \cdot 10^{-5}$

^{c} $m_G, m_S, r = 1$, $r_{history} = 50$, $\mu = 10^{-4}$

Table C: Effective Sample Sizes of the model parameters calculated for a various number of introductions. Results are the mean of 75 chains, i.e. 3 coalescent rates per number of introductions and 25 outbreaks per parameter set.

Simulated number of introductions	Parameters				
	μ	m_G	m_S	r	$r_{history}$
1	3565	6590	1187	512	1629
2	623	5426	907	496	1191
5	183	5399	1082	546	1314
10	411	3154	699	491	1583
15	556	1660	491	593	1479
20	373	420	227	635	1067

Table D: Effective Sample Sizes (ESS) of the model parameters for analyzing a SARS-CoV-2 outbreak in mink farms in the Netherlands.

Parameters	ESS
log-likelihood	633
μ	710
mS	205
r	376
r_{history}	232

Table E: Clustering mink farms infected with SARS-CoV-2 based on a 2 SNP or 3 SNP cutoff. The coloring of the farms in the third column represents the 13 introductions found. The black-colored farms are single-case transmission chains.

3 SNP cutoff	2 SNP cutoff	Farms
A	A1	1, 4, 8b, 11, 12, 13, 16, 20
	A2	33, 35, 36, 40, 42, 43, 44 47, 48, 50, 51, 52, 53, 54, 56 57, 58, 59, 60, 61, 63
	A3	55, 62
	Single farms	21, 27, 49, 68
C	C1	7, 9, 31
	C2	17, 23, 26, 29, 32, 34, 39, 41 45, 46
	Single farms	6, 14, 22, 26
D	D1	10, 15, 18, 19, 25, 28
	Single farms	2, 8a
Single farms		3, 5, 38
