# Supplementary material for: A Bayesian inference method to estimate transmission trees with multiple introductions; applied to SARS-CoV-2 in Dutch mink farms
Source: PLoS Comput Biol. 2023 Nov 27;19(11):e1010928. doi: 10.1371/journal.pcbi.1010928 (PMC10703282; doi:10.1371/journal.pcbi.1010928)
Supplement: S1 Methods — (PDF) [file pcbi.1010928.s002.pdf]

Supplementary Methods

Part 1: The posterior probability of the transmission tree and phylogenetic tree.

Definitions:

- n = outbreak size
- **S** with elements S_i = sampling times per host $i \in [1,n]$
- **G** with elements G_i = genome sequences per host $i \in [1,n]$, each consisting of L nucleotides *A*, *C*, *G*, *T*, or – if unknown – *N*
  - D_{ij} = number of different nucleotides between hosts *i* and *j*, not counting *N*'s
  - $D_{\max}$ = maximum D_{ij} for all host pairs *i* and *j*
  - N_{ij} = number of nucleotide positions with *N* in host *i* and/or *j*
  - $d_{ij} = D_{ij} + N_{ij}\, D_{\max} / L$ = sequence distance between hosts *i* and *j*
- **I** with elements I_i = infection times per host $i \in [1,n]$.
- **M** with elements M_i = infectors per host $i \in [1,n]$
- P is the phylogenetic tree, being a set of numbered nodes x:
  - $x \in [1,n]$ are the sampling nodes, corresponding to hosts *i*
  - $x \in [n+1, 2n-1]$ are the coalescent nodes
  - $x \in [2n, 3n-1]$ are the transmission nodes, with node *x* corresponding to the infection of host $i = x + 1 - 2n$
- Further, we define t_x as the time of node *x*, v_x as the ancestor node of node *x*, and h_x as the host in which node *x* resides (transmission nodes assigned to the infector, as the value of *x* already defines the infectee). Among all t_x, $t_{\min}$ is the lowest and $t_{\max}$ is the highest value.
- $mrca_{x,y}$ = MRCA (most recent common ancestor) of nodes *x* and *y*
- P_i are the phylogenetic trees within each host (including history host 0): $P_i = \{x \mid h_x = i\}$, for which we further define $\tau_x = t_x - t_{h_x + 2n - 1}$ as the time of the node since infection of the host. In the history host, all $\tau_x = \infty$
- **θ** = parameter vector, with parameters for the introduction rate, the generation time and sampling time distributions, the parameters describing the within-host coalescent model, and the mutation rate
  - λ_{intro} = rate of new introductions
  - a_G, m_G = shape parameter and mean of gamma-distributed generation time
  - a_S, m_S = shape parameter and mean of gamma-distributed sampling time
  - r = slope of within-host pathogen growth model
  - $r_{history}$ = pathogen population size in the history host
  - μ = mutation rate

notation

- $d_{\Gamma(a,m)}; p_{\Gamma(a,m)}$ are the density and cumulative density of a Gamma distribution with shape parameter *a* and mean *m*
- $\Pr(.)$ is a probability or probability density
- $u(\tau)$ is the heaviside step function, equal to 0 if $\tau < 0$, and equal to 1 if $\tau \geq 0$

The complete posterior probability

$$\Pr\left(\mathbf{I},\mathbf{M},P,\boldsymbol{\theta}\middle|\mathbf{S},\mathbf{G}\right)\propto\Pr\left(\mathbf{S},\mathbf{G}\middle|\mathbf{I},\mathbf{M},P,\boldsymbol{\theta}\right)\cdot\Pr\left(\mathbf{I},\mathbf{M},P,\boldsymbol{\theta}\right)$$
$$=\Pr\left(\mathbf{S},\mathbf{G}\middle|\mathbf{I},\mathbf{M},P,\boldsymbol{\theta}\right)\cdot\Pr\left(\mathbf{I},\mathbf{M},P\middle|\boldsymbol{\theta}\right)\cdot\Pr\left(\boldsymbol{\theta}\right)$$
$$=\Pr\left(\mathbf{S},\mathbf{G},\mathbf{I},\mathbf{M},P\middle|\boldsymbol{\theta}\right)\cdot\Pr\left(\boldsymbol{\theta}\right)$$
$$=\Pr\left(\mathbf{G}\middle|\mathbf{S},\mathbf{I},\mathbf{M},P,\boldsymbol{\theta}\right)\cdot\Pr\left(P\middle|\mathbf{S},\mathbf{I},\mathbf{M},\boldsymbol{\theta}\right)\cdot\Pr\left(\mathbf{S}\middle|\mathbf{I},\mathbf{M},\boldsymbol{\theta}\right)\cdot\Pr\left(\mathbf{I},\mathbf{M}\middle|\boldsymbol{\theta}\right)\cdot\Pr\left(\boldsymbol{\theta}\right)$$
$$=\Pr\left(\mathbf{G}\middle|P,\boldsymbol{\theta}\right)\cdot\Pr\left(P\middle|\mathbf{S},\mathbf{I},\mathbf{M},\boldsymbol{\theta}\right)\cdot\Pr\left(\mathbf{S}\middle|\mathbf{I},\boldsymbol{\theta}\right)\cdot\Pr\left(\mathbf{I},\mathbf{M}\middle|\boldsymbol{\theta}\right)\cdot\Pr\left(\boldsymbol{\theta}\right)$$

All steps until the last follow from standard probability rules. In the last step, dependencies are removed that do not exist in the model. In the last line, the first four terms are likelihood terms, that will be elaborated below. The last term is the prior.

Likelihood #1 for the genetic data

We assume a Jukes-Cantor substitution model with mutation rate μ:

$$\Pr\left(\mathbf{G}\middle|P,\boldsymbol{\theta}\right)=\prod_{loci}\sum_{\{A,C,T,G\}^{3n-1}}\prod_{x}\left(\tfrac{1}{4}-\tfrac{1}{4}\exp\left(-\mu\left(t_x-t_{v_x}\right)\right)\right)^{\mathrm{I}_{mut}(1-\mathrm{N})}\cdot\left(\tfrac{1}{4}+\tfrac{3}{4}\exp\left(-\mu\left(t_x-t_{v_x}\right)\right)\right)^{(1-\mathrm{I}_{mut})(1-\mathrm{N})}$$

For each locus, for each possible assignment at each internal node (coalescent and transmission), for all branches in the tree indicated by the end node *x*, the probability of a mutation is calculated where I_{mut} indicates if a mutation occurred on that branch and N indicates if the branch ends in a tip without observed nucleotide ('n' in the sequence data). This likelihood can be calculated using Felsenstein's pruning algorithm [1]. Note that in the above formulation the actual rate of nucleotide *change* is 0.75μ, as a mutation gives rise to any of the four nucleotides.

Likelihood #2 for the phylogenetic tree

The likelihood for the complete phylogenetic tree is a product of the likelihoods of trees in individual hosts:

$$\Pr\left(P\middle|\mathbf{S},\mathbf{I},\mathbf{M},\boldsymbol{\theta}\right)=\Pr\left(P_0\middle|\mathbf{I},\mathbf{M},\boldsymbol{\theta}\right)\prod_{i>0}\Pr\left(P_i\middle|S_i,\mathbf{I},\mathbf{M},\boldsymbol{\theta}\right)$$

The dependency on the complete vectors **I** and **M** remains, because these determine the transmission nodes with host *i* as infector.

We define the number of lineages in history host 0 at time *t*, and in all other hosts *i* at time τ since infection as

$$L_0(t) = \sum_{x|x\in P_0 \cap n<x<2n} u(t-t_x) - \sum_{x|x\in P_0 \cap x\geq 2n} u(t-t_x) - u(t-t_i)$$
$$L_{i,i>0}(\tau) = 1 + \sum_{x|x\in P_i \cap n<x<2n} u(\tau-\tau_x) - \sum_{x|x\in P_i \cap x\geq 2n} u(\tau-\tau_x) - u(\tau-\tau_i),$$

where $u(\tau)$ is the heaviside step function, i.e. $u(\tau)=0$ if $\tau<0$ and $u(\tau)=1$ if $\tau\geq 0$, adding 1 at infection and at each coalescent node, and subtracting 1 at each transmission node and at sampling .

The within-host dynamics $w(\tau,r)$ describes the product of pathogen generation time and effective population size, the inverse of which determines the coalescent rate. As a general form, we choose for hosts 1…*n*

$$w(\tau,r) = r\tau,$$

which automatically gives a bottleneck of size 1 (because the coalescent rate $\to\infty$ as $\tau\downarrow 0$), allows for most coalescent nodes close to the time of infection (high *r*) or close to the transmission nodes (low *r*). In history host 0, we choose a constant population size

$$w = r_{history}$$

Thus, the likelihood for the phylogenetic tree in host *i* becomes

$$\Pr\left(P_0|\mathbf{I},\mathbf{M},\boldsymbol{\theta}\right) = \exp\left(-\int_{-\infty}^{\infty}\binom{L_0(t)}{2}\frac{1}{r_{history}}dt\right)\prod_{x|x\in P_0\cap n<x<2n}\frac{1}{r_{history}}$$
$$\Pr\left(P_{i,i>0}|S_i,\mathbf{I},\mathbf{M},\boldsymbol{\theta}\right) = \exp\left(-\int_{0}^{\infty}\binom{L_i(\tau)}{2}\frac{1}{w(\tau,r)}d\tau\right)\prod_{x|x\in P_i\cap n<x<2n}\frac{1}{w(\tau_x,r)},$$

with $\binom{0}{2}\equiv\binom{1}{2}\equiv 0$.

Likelihood #3 for the sampling intervals

The sampling intervals are assumed to follow a gamma distribution with shape parameter a_S and with mean m_S. The likelihood is the product of densities for all sampling times:

$$\Pr\left(\mathbf{S}|\mathbf{I},\boldsymbol{\theta}\right) = \prod_i d_{\Gamma(a_S,m_S)}\left(S_i - I_i\right)$$

Likelihood #4 for infection times and infectors

The index cases following the initial index case are assumed to be generated at a rate λ_{intro}, between the first infection time $t_{\min}$ until the last sampling time $t_{\max}$. Subsequent cases are generated with a reproduction number of 1, with the generation intervals following a gamma distribution with shape parameter a_G and with mean m_G. The likelihood is the product of densities by which all cases were generated:

$$\Pr\left(\mathbf{I},\mathbf{M}\middle|\boldsymbol{\theta}\right)=\Pr\left(\mathbf{I}\middle|\mathbf{M},\boldsymbol{\theta}\right)\cdot\Pr\left(\mathbf{M}\middle|\boldsymbol{\theta}\right)=\tfrac{1}{\lambda_{intro}}\exp\left(-\lambda_{intro}\left(t_{\max}-t_{\min}\right)\right)\prod_{i|M_i=0}\lambda_{intro}\prod_{i|M_i>0}d_{\Gamma(a_G,m_G)}\left(I_i-I_{M_i}\right)$$

Prior distributions

The model has 8 parameters: introduction rate λ_{intro}, mutation rate μ, within-host growth rate r, history host population size $r_{history}$, sampling interval distribution parameters a_S and m_S, and generation interval distribution parameters a_G and m_G. In the current implementation, a_S and a_G are not estimated but chosen before the analysis. The prior distributions for the other parameters are

- $\lambda_{intro} \sim \Gamma\left(a_{0,\lambda}, m_{0,\lambda}\right)$. When using an uninformative prior, we set $a_{0,\lambda}=1$ and $m_{0,\lambda}=1$.
- $\mu \sim N\left(\mu_{0,\mu}, \sigma_{0,\mu}\right)$, only for positive μ. When using an uninformative prior, we set $\mu_{0,\mu}=0$ and $\sigma_{0,\mu}=10$.
- $m_S \sim D\left(\mu_{0,S}, \sigma_{0,S}\right)$, a prior distribution with mean $\mu_{0,S}$ and standard deviation $\sigma_{0,S}$, which is translated (see box below) into a Gamma-distributed prior for the rate parameter b_S of the sampling interval Gamma distribution:
  $b_S \sim \Gamma\left(a_{0,S}=2+\mu_{0,S}^2/\sigma_{0,S}^2, b_{0,S}=\left(\mu_{0,S}/a_S\right)\cdot\left(1+\mu_{0,S}^2/\sigma_{0,S}^2\right)\right)$. Here, $a_{0,S}$ is the shape and $b_{0,S}$ is the rate. When using an uninformative prior, we set $\mu_{0,S}=1$ and $\sigma_{0,S}=\infty$.
- $m_G \sim D\left(\mu_{0,G}, \sigma_{0,G}\right)$, as for m_S.
- $r \sim \Gamma\left(a_{0,r}, m_{0,r}\right)$. When using an uninformative prior, we set $a_{0,r}=3$ and $m_{0,r}=1$.
- $r_{history} \sim \Gamma\left(a_{0,r_{history}}, m_{0,r_{history}}\right)$. When using an uninformative prior, we set $a_{0,r_{history}}=1$ and $m_{0,r_{history}}=100$.

> We have a_S fixed, and define $b_S \sim \Gamma\left(a_{0,S}, b_{0,S}\right)$, such that
>
> 1. $E\left(a_S/b_S\right)=\mu_{0,S} \Leftrightarrow E\left(1/b_S\right)=\mu_{0,S}/a_S$
> 2. $E\left(\left(\mu_{0,S}-a_S/b_S\right)^2\right)=\sigma_{0,S}^2 \Leftrightarrow E\left(\left(\mu_{0,S}/a_S-1/b_S\right)^2\right)=\sigma_{0,S}^2/a_S^2$
>
> Now, we wish to calculate $a_{0,S}$ and $b_{0,S}$.
> If the density of rate b_S is $d_\Gamma\left(b_S\right)$, then the density of scale $\theta_S=1/b_S$ is $d_\Gamma\left(\theta_S\right)/\theta_S^2$. This is an inverse Gamma distribution, of which the mean and variance can be filled into the equations above:
>
> 1. $\mu_{0,S}/a_S=\dfrac{b_{0,S}}{a_{0,S}-1}$
> 2. $\sigma_{0,S}^2/a_S^2=\dfrac{b_{0,S}^2}{\left(a_{0,S}-1\right)^2\left(a_{0,S}-2\right)}$
>
> Solving these equations for $a_{0,S}$ and $b_{0,S}$ gives the prior distribution for b_S.

Part 2: Updating steps in the MCMC chain.

The mean sampling and generation intervals are directly sampled from their posteriors. For the other parameters in $\boldsymbol{\theta}$ and unobserved variables $\mathbf{Z}$ = {**I, M,** P}, updating is always done by a Metropolis-Hastings step:

- proposing new values with proposal density $G(\boldsymbol{\theta}'|\boldsymbol{\theta})$ or $H(\mathbf{Z}'|\mathbf{Z},\mathbf{S},\boldsymbol{\theta})$, respectively
- accepting the new values with probability α,

$$\alpha = \min\left[1, \frac{\Pr(\mathbf{S},\mathbf{G},\mathbf{Z}|\boldsymbol{\theta}')\cdot\Pr(\boldsymbol{\theta}')\cdot G(\boldsymbol{\theta}|\boldsymbol{\theta}')}{\Pr(\mathbf{S},\mathbf{G},\mathbf{Z}|\boldsymbol{\theta})\cdot\Pr(\boldsymbol{\theta})\cdot G(\boldsymbol{\theta}'|\boldsymbol{\theta})}\right]$$

or

$$\alpha = \min\left[1, \frac{\Pr(\mathbf{S},\mathbf{G},\mathbf{Z}'|\boldsymbol{\theta})\cdot\Pr(\boldsymbol{\theta})\cdot H(\mathbf{Z}|\mathbf{Z}',\mathbf{S},\boldsymbol{\theta})}{\Pr(\mathbf{S},\mathbf{G},\mathbf{Z}|\boldsymbol{\theta})\cdot\Pr(\boldsymbol{\theta})\cdot H(\mathbf{Z}'|\mathbf{Z},\mathbf{S},\boldsymbol{\theta})}\right] = \min\left[1, \frac{\Pr(\mathbf{S},\mathbf{G},\mathbf{Z}'|\boldsymbol{\theta})\cdot H(\mathbf{Z}|\mathbf{Z}',\mathbf{S},\boldsymbol{\theta})}{\Pr(\mathbf{S},\mathbf{G},\mathbf{Z}|\boldsymbol{\theta})\cdot H(\mathbf{Z}'|\mathbf{Z},\mathbf{S},\boldsymbol{\theta})}\right],$$

respectively

Updating the mean sampling and generation intervals

The parameters m_S and m_G are updated by direct sampling from the posterior distributions of rate parameters b_S and b_G and calculating $m_S = a_S/b_S$ and $m_G = a_G/b_G$. These posterior distributions are:

$$b_S \sim \Gamma\left(a_{post,S} = a_{0,S} + n\cdot a_S, b_{post,S} = b_{0,S} + \sum_i S_i - I_i\right)$$

$$b_G \sim \Gamma\left(a_{post,G} = a_{0,G} + \left(\sum_{i|M_i\neq 0} 1\right)\cdot a_G, b_{post,G} = b_{0,G} + \sum_{i|M_i\neq 0} I_i - I_{M_i}\right)$$

Updating other parameters in $\boldsymbol{\theta}$

Proposal

A new value ζ' (being $\lambda_{history}'$, μ', r', or $r_{history}'$) is proposed as

$$\log(\zeta') \sim N\left(\log(\zeta), \sigma_\zeta^2\right)$$

Thus, the proposal density of ζ' is

$$G(\zeta'|\zeta) = d_N\left(\log(\zeta') - \log(\zeta), \sigma_\zeta^2\right)/\zeta'$$

The proposal variance σ_ζ^2 is calculated from the available data as follows:

- For $\log(\mu)$, the posterior density is proportional to the likelihood (and the prior). Although the actual likelihood calculation is done with Felsenstein's pruning algorithm [1], μ is the rate of a Poisson process, so it should be possible to express it in terms of a number of realizations and the total exposure time. The number of realizations *x* is the number of

mutations, i.e. the parsimony of the phylogenetic tree; the exposure time *D* is the sum of all branch length multiplied by the sequence length. By expressing the likelihood of $\log(\mu)$ in terms of *x* and *D*, and normalizing it to get a proper distribution, the variance of this distribution can be calculated as $\psi_1(x)$, which is the trigamma function. Optimal proposals in a Metropolis-Hastings step have a variance of 2.38^2 times the target variance [2]. Assuming that the posterior phylogenetic trees reach maximum parsimony, the proposal variance is set to $\sigma_\mu^2 = 2.38^2\psi_1(x)$, with *x* the number of SNPs in the dataset.

- For $\log(r)$, a similar reasoning is used, because $1/r$ is proportional to the rate of a Poisson process as well. Here, the number of realizations *x* is the number of coalescent nodes, which is equal to *n* – 1 (or possibly smaller, in the case of multiple introdutions). So, $\sigma_r^2 = 2.38^2\psi_1(n-1)$.
- For $\log(r_{history})$, the same variance as for $\log(r)$ is used, although the number of coalescent nodes in the history host will be smaller in most cases.
- For $\log(\lambda_{intro})$, a similar reasoning is not possible because the number of introductions is not known beforehand. The variance will be calculated based on the number of introductions n_{intro} at initialisation: $\sigma_\lambda^2 = 2.38^2\psi_1(n_{intro})$

Acceptance probability

The acceptance probability is

$$\min\left[1, \frac{\Pr(\mathbf{S},\mathbf{G},\mathbf{Z}|\boldsymbol{\theta}')\cdot\Pr(\boldsymbol{\theta}')\cdot G(\boldsymbol{\theta}|\boldsymbol{\theta}')}{\Pr(\mathbf{S},\mathbf{G},\mathbf{Z}|\boldsymbol{\theta})\cdot\Pr(\boldsymbol{\theta})\cdot G(\boldsymbol{\theta}'|\boldsymbol{\theta})}\right]$$

$$=\min\left[1, \frac{\Pr(\mathbf{S},\mathbf{G},\mathbf{Z}|\boldsymbol{\theta}')\cdot\Pr(\zeta')\cdot d_N\left(\log(\zeta)-\log(\zeta'),\sigma_\zeta^2\right)/\zeta}{\Pr(\mathbf{S},\mathbf{G},\mathbf{Z}|\boldsymbol{\theta})\cdot\Pr(\zeta)\cdot d_N\left(\log(\zeta')-\log(\zeta),\sigma_\zeta^2\right)/\zeta'}\right]$$

$$=\min\left[1, \frac{\Pr(\mathbf{S},\mathbf{G},\mathbf{Z}|\boldsymbol{\theta}')\cdot\Pr(\zeta')/\zeta}{\Pr(\mathbf{S},\mathbf{G},\mathbf{Z}|\boldsymbol{\theta})\cdot\Pr(\zeta)/\zeta'}\right]$$

Updating the transmission tree and phylogenetic tree

Proposals

The unobserved variables are updated in small sets at once. Three types of proposal are distinguished when analysing outbreaks with a single introduction: the first in which both transmission tree and phylogenetic tree are changed, the second in which only the transmission tree is changed, and the third in which only the phylogenetic tree is changed. In the R package the user can choose the probabilities with which these three proposals are used; the default is 80%-20%-0% for the three types, respectively.

When using the method allowing multiple introductions, the first proposal type is used for updates within subtrees (all cases following from one index case), a fourth proposal type is used for changes across subtrees, and a fifth proposal for the history host. For the hosts other than the history host, the user can choose the probabilities with which the two proposals are used; the default is 50%-50%. All four proposals take a single focal host *i*; proposals 1, 2 and 4 start with step 1:

1. propose a new infection time I_i' by sampling $T \sim \Gamma(a_P, m_S)$ from a Gamma distribution with shape = $a_P = \frac{2}{3}a_S$ and mean = m_S, and calculating $I_i' = S_i - T$

Then, the following decision trees determine the subsequent proposal steps and acceptance probabilities, laid out in proposal paths A-M below. In some cases, the proposal is immediately rejected if no reverse proposal exists:

Proposal 1: changing phylogenetic and transmission trees (within a subtree).

Q1: is host *i* index case?

{Q1=Y}Q2: is $I_i' < \min(\{I_j | M_j = i\}) \rightarrow$ is I_i' before host *i*'s first transmission node?

{Q12=YY} **follow proposal path A (Fig M1A)**

{Q12=YN}Q3: is $I_i' < \{I_j | M_j = i\}_{(2)} \rightarrow$ is I_i' before host *i*'s second transmission node?

(Yes, if no second transmission node exists)

{Q123=YNY} **follow proposal path B (Fig M1B)**

{Q123=YNN} **follow proposal path C (Fig M1C)**

{Q1=N}Q2: is $I_i' < \min(\mathbf{I}) \rightarrow$ is I_i' before infection of the index case (in *i*'s subtree)?

{Q12=NY} **follow proposal path D (Fig M1D)**

{Q12=NN}Q3: is $I_i' < \min(\{I_j | M_j = i\}) \rightarrow$ is I_i' before host *i*'s first transmission node?

(Yes, if no transmission node exists)

{Q123=NNY} **follow proposal path E (Fig M1E)**

{Q123=NNN} **follow proposal path F (Fig M1F)**

Proposal 2: changing transmission tree, but not phylogenetic tree.

Q1: is host *i* index case?

{Q1=Y}Q2: is $I_i' < \min(\{\tau_x | h_x = i\}) \rightarrow$ is I_i' before host *i*'s first coalescent node?

{Q12=YY} **follow proposal path G**

{Q12=YN} **reject**

{Q1=N}Q2: is $I_i' > mrca_{i,M_i} \rightarrow$ is I_i' after the *mrca* of the samplings in hosts *i* and M_i?

{Q12=NY} **follow proposal path H (Fig M2A)**

{Q12=NN}: new proposal step:

2. propose I_{M_i}' by sampling $T \sim \Gamma(a_P, m_S)$ and calculating $I_{M_i}' = S_{M_i} - T$

Q3: is $I_{M_i}' > mrca_{i,M_i} \rightarrow$ is I_{M_i}' after the *mrca* of host *i* and its infector?

{Q123=NNY}Q4: is $M_{M_i} = 0 \rightarrow$ is host *i*'s infector the index case?

{Q1234=NNYY} **follow proposal path I (Fig M2B)**
{Q1234=NNYN}Q5: is I_i' after the *mrca* of the samplings in hosts *i* and M_{M_i} ?
{Q12345=NNYNY} **follow proposal path J (Fig M2C)**
{Q12345=NNYNN} **reject**
{Q123=NNN} **reject**

Proposal 3: changing phylogenetic tree topology only.
follow proposal path K

Proposal 4: changing phylogenetic and transmission trees, across all subtrees
Q1: is $I_i' < \min\left(\left\{I_j \middle| M_j = i\right\}\right) \rightarrow$ is I_i' before host *i*'s first transmission node?
{Q1=Y} **follow proposal path E (Fig M1E)**
{Q1=N} **reject**

Proposal 5: changing the phylogenetic tree of the history host.
follow proposal path L

Proposal path A {Proposal 1, Q12=YY}

Situation
Host *i* is index case, and I_i' is before its first transmission node.

Proposed changes to transmission tree

1. Topological changes: none
2. Infection time changes:
   a. I_i changes to I_i'

Proposal steps

2. propose a new tree P_i' by simulating the within-host coalescent model

Proposal distribution
The proposal distribution for I_i' and P_i' is

$$\begin{aligned} H\left(I_i',P_i'\middle|\mathbf{I},\mathbf{M},P,\mathbf{S},\boldsymbol{\theta}\right) &= \Pr\left(P_i'\middle|I_i',\mathbf{I},\mathbf{M},P,\mathbf{S},\boldsymbol{\theta}\right)\cdot\Pr\left(I_i'\middle|\mathbf{I},\mathbf{M},P,\mathbf{S},\boldsymbol{\theta}\right) \\ &= \Pr\left(P_i'\middle|\mathbf{I}',\mathbf{M},S_i,\boldsymbol{\theta}\right)\cdot\Pr\left(I_i'\middle|S_i\right) \\ &= \Pr\left(P_i'\middle|\mathbf{I}',\mathbf{M},S_i,\boldsymbol{\theta}\right)\cdot d_{\Gamma(a_P,m_S)}\left(S_i - I_i'\right) \end{aligned}$$

Acceptance probability
The acceptance probability (removing the dependency on θ after the first line, for readability) is

$$\begin{aligned} &\min\left[1,\frac{\Pr\left(\mathbf{S},\mathbf{G},\mathbf{Z}'\middle|\boldsymbol{\theta}\right)\times H\left(\mathbf{Z}\middle|\mathbf{Z}',\mathbf{S},\boldsymbol{\theta}\right)}{\Pr\left(\mathbf{S},\mathbf{G},\mathbf{Z}\middle|\boldsymbol{\theta}\right)\times H\left(\mathbf{Z}'\middle|\mathbf{Z},\mathbf{S},\boldsymbol{\theta}\right)}\right] \\ &=\min\left[1,\frac{\Pr\left(\mathbf{G}\middle|P'\right)\cdot\Pr\left(P'\middle|\mathbf{S},\mathbf{I}',\mathbf{M}\right)\cdot\Pr\left(\mathbf{S}\middle|\mathbf{I}'\right)\cdot\Pr\left(\mathbf{I}',\mathbf{M}\right)\times\Pr\left(P_i\middle|S_i,\mathbf{I},\mathbf{M}\right)\cdot d_{\Gamma(a_P,m_S)}\left(S_i-I_i\right)}{\Pr\left(\mathbf{G}\middle|P\right)\cdot\Pr\left(P\middle|\mathbf{S},\mathbf{I},\mathbf{M}\right)\cdot\Pr\left(\mathbf{S}\middle|\mathbf{I}\right)\cdot\Pr\left(\mathbf{I},\mathbf{M}\right)\times\Pr\left(P_i'\middle|S_i,\mathbf{I}',\mathbf{M}\right)\cdot d_{\Gamma(a_P,m_S)}\left(S_i-I_i'\right)}\right] \\ &=\min\left[1,\frac{\Pr\left(\mathbf{G}\middle|P'\right)\cdot\Pr\left(S_i\middle|I_i'\right)\cdot\Pr\left(\mathbf{I}',\mathbf{M}\right)\cdot d_{\Gamma(a_P,m_S)}\left(S_i-I_i\right)}{\Pr\left(\mathbf{G}\middle|P\right)\cdot\Pr\left(S_i\middle|I_i\right)\cdot\Pr\left(\mathbf{I},\mathbf{M}\right)\cdot d_{\Gamma(a_P,m_S)}\left(S_i-I_i'\right)}\right] \end{aligned}$$

Reverse proposal
This sampling step can be reversed by proposing through (the same) proposal path A, with the original infection time I_i proposed for the same focal host *i* (going back in Fig M1A).

Proposal path B {Proposal 1, Q123=YNY}

Situation
Host *i* is index case and I_i' is sampled after its first transmission node (infecting host *j*), but before its second transmission node, if there is any.

Proposed changes to transmission tree

1. Topological changes:
    a. host *i* gets a new infector
    b. host *j* becomes index case
2. Infection time change:
    a. I_i changes to I_i'

Proposal steps

2. propose a new infector M_i' from the proposal distribution $F_j(I_i')$, which is the probability to select infector *j* at time I_i' in the outbreak. The proposal distribution is given by

$$F_j(t) = \frac{u(t - I_j)\cdot\left(d_{\Gamma(a_G, b_G)}(t - I_j) + 1/d_{ij}\right)}{\sum_k \left[u(t - I_k)\cdot\left(d_{\Gamma(a_G, b_G)}(t - I_k) + 1/d_{ij}\right)\right]},$$

    so the probability to select infector *j* is proportional to the density of the generation time plus a weight $1/d_{ij}$, with d_{ij} equal to the sequence distance between isolates from hosts *i* and *j* (see list of definitions). This proposal distribution gives a sampling weight to possible infectors based on their infection time and related to the genetic distance between isolates.
3. host *j* becomes the index case: $M_j' = 0$
4. bookkeeping: make corresponding changes in the nodes and move one of the coalescent nodes from host *i* to host M_i'
5. propose new trees P_i' and $P_{M_i'}'$ by simulating the within-host coalescent model

Proposal distribution
The proposal distribution for I_i', M_i', M_j', P_i', and $P_{M_i'}'$ is

$$\begin{aligned}
H\left(I_i', M_i', M_j', P_i', P_{M_i'}' \middle| \mathbf{I}, \mathbf{M}, P, \mathbf{S}, \boldsymbol{\theta}\right)
&= \Pr\left(P_i', P_{M_i'}' \middle| I_i', M_i', M_j', \mathbf{I}, \mathbf{M}, P, \mathbf{S}, \boldsymbol{\theta}\right)\cdot \\
&\quad \Pr\left(M_i', M_j' \middle| I_i', \mathbf{I}, \mathbf{M}, P, \mathbf{S}, \boldsymbol{\theta}\right)\cdot \\
&\quad \Pr\left(I_i' \middle| \mathbf{I}, \mathbf{M}, P, \mathbf{S}, \boldsymbol{\theta}\right) \\
&= \Pr\left(P_i' \middle| \mathbf{I}', \mathbf{M}', S_i, \boldsymbol{\theta}\right)\cdot \Pr\left(P_{M_i'}' \middle| \mathbf{I}', \mathbf{M}', S_j, \boldsymbol{\theta}\right)\cdot \Pr\left(M_i' \middle| \mathbf{I}', \boldsymbol{\theta}\right)\cdot \Pr\left(M_j' \middle| \mathbf{I}', \boldsymbol{\theta}\right)\cdot \Pr\left(I_i' \middle| S_i, \boldsymbol{\theta}\right) \\
&= \Pr\left(P_i', P_{M_i'}' \middle| \mathbf{I}', \mathbf{M}', \mathbf{S}, \boldsymbol{\theta}\right)\cdot F_{M_i'}(I_i')\cdot d_{\Gamma(a_P, m_S)}(S_i - I_i')
\end{aligned}$$

Here, $\Pr\left(M_j'\middle|\ldots\right)=1$ because it follows automatically from the proposed I_i'.

Acceptance probability

The acceptance probability (removing the dependency on θ after the first line, for readability) is

$$\min\left[1,\frac{\Pr\left(\mathbf{S},\mathbf{G},\mathbf{Z}'\middle|\boldsymbol{\theta}\right)\times H\left(\mathbf{Z}\middle|\mathbf{Z}',\mathbf{S},\boldsymbol{\theta}\right)}{\Pr\left(\mathbf{S},\mathbf{G},\mathbf{Z}\middle|\boldsymbol{\theta}\right)\times H\left(\mathbf{Z}'\middle|\mathbf{Z},\mathbf{S},\boldsymbol{\theta}\right)}\right]$$
$$=\min\left[1,\frac{\Pr\left(\mathbf{G}\middle|P'\right)\cdot\Pr\left(P'\middle|\mathbf{S},\mathbf{I}',\mathbf{M}'\right)\cdot\Pr\left(\mathbf{S}\middle|\mathbf{I}'\right)\cdot\Pr\left(\mathbf{I}',\mathbf{M}'\right)\times\Pr\left(P_i,P_{M_i'}\middle|\mathbf{S},\mathbf{I},\mathbf{M}\right)\cdot d_{\Gamma(a_P,m_S)}\left(S_i-I_i\right)}{\Pr\left(\mathbf{G}\middle|P\right)\cdot\Pr\left(P\middle|\mathbf{S},\mathbf{I},\mathbf{M}\right)\cdot\Pr\left(\mathbf{S}\middle|\mathbf{I}\right)\cdot\Pr\left(\mathbf{I},\mathbf{M}\right)\times\Pr\left(P_i',P_{M_i'}'\middle|\mathbf{S},\mathbf{I}',\mathbf{M}'\right)\cdot F_{M_i'}\left(I_i'\right)\cdot d_{\Gamma(a_P,m_S)}\left(S_i-I_i'\right)}\right]$$
$$=\min\left[1,\frac{\Pr\left(\mathbf{G}\middle|P'\right)\cdot\Pr\left(S_i\middle|I_i'\right)\cdot\Pr\left(\mathbf{I}',\mathbf{M}'\right)\cdot d_{\Gamma(a_P,m_S)}\left(S_i-I_i\right)}{\Pr\left(\mathbf{G}\middle|P\right)\cdot\Pr\left(S_i\middle|I_i\right)\cdot\Pr\left(\mathbf{I},\mathbf{M}\right)\cdot F_{M_i'}\left(I_i'\right)\cdot d_{\Gamma(a_P,m_S)}\left(S_i-I_i'\right)}\right]$$

Reversal

Reversal is possible through proposal path D. Fig M1D shows the reverse proposal path D from the original path B in Fig M1B.

Proposal path C {Proposal 1, Q123=YNN}

Situation

Host *i* is the index case and I_i' is sampled after its second transmission node. Host *j* is the first secondary case.

Proposed changes to transmission tree

1. Topological changes:
    a. host *j* becomes index case, transmitting to host *i*
    b. with 50% probability: the other secondary cases of hosts *i* and *j* are exchanged
2. Infection time change:
    a. the sampled I_i' is discarded; instead, the infection times of hosts *i* and *j* are switched

Proposal steps

2. discard the sampled infection time and switch infection times by proposing $I_i'=I_j$ and $I_j'=I_i$
3. switch role by proposing $M_i'=j$ and $M_j'=0$
4. bookkeeping: make corresponding changes in the nodes and move one of the coalescent nodes from host *i* to host *j*
5. with 50% probability: for all $\left\{k\middle|M_k=i\wedge k\neq j\right\}$, propose $M_k'=j$; for all $\left\{k\middle|M_k=j\right\}$, propose $M_k'=i$; finish by bookkeeping: swap all transmission and coalescent nodes between P_i and P_j
6. propose new trees P_i' and P_j' by simulating the within-host coalescent model

Proposal distribution

The proposal distribution for I_i', I_j', **M**, P_i', and P_j' is

$$
\begin{aligned}
&H\left(I_i',I_j',\mathbf{M}',P_i',P_j'\middle|\mathbf{I},\mathbf{M},P,\mathbf{S},\boldsymbol{\theta}\right)\\
&\qquad=\Pr\left(P_i',P_j'\middle|I_i',I_j',\mathbf{M}',\mathbf{I},\mathbf{M},P,\mathbf{S},\boldsymbol{\theta}\right)\cdot\\
&\qquad\quad\Pr\left(\mathbf{M}'\middle|I_i',I_j',\mathbf{I},\mathbf{M},P,\mathbf{S},\boldsymbol{\theta}\right)\cdot\\
&\qquad\quad\Pr\left(I_i',I_j'\middle|\mathbf{I},\mathbf{M},P,\mathbf{S},\boldsymbol{\theta}\right)\\
&\qquad=\Pr\left(P_i'\middle|\mathbf{I}',\mathbf{M}',S_i,\boldsymbol{\theta}\right)\cdot\Pr\left(P_j'\middle|\mathbf{I}',\mathbf{M}',S_j,\boldsymbol{\theta}\right)\cdot\\
&\qquad\quad\Pr\left(\mathbf{M}'\middle|\mathbf{I}',\boldsymbol{\theta}\right)\cdot\Pr\left(I_j'\middle|I_i',\boldsymbol{\theta}\right)\cdot\Pr\left(I_i'\middle|\mathbf{I},S_i,\boldsymbol{\theta}\right)\\
&\qquad=\Pr\left(P_i',P_j'\middle|\mathbf{I}',\mathbf{M}',\mathbf{S},\boldsymbol{\theta}\right)\cdot 0.5\cdot p_{\Gamma(a_P,m_S)}\left(S_i-I_j\right)
\end{aligned}
$$

Here, $\Pr\left(\mathbf{M}'\middle|\mathbf{M},\boldsymbol{\theta}\right)=0.5$ because of the two possible rearrangements of infectees that follow automatically from the proposed I_i', and $\Pr\left(I_j'\middle|I_i',\boldsymbol{\theta}\right)=1$ because it follows automatically from the proposed I_i'. In the last step, $\Pr\left(I_i'\middle|\mathbf{I},S_i,\boldsymbol{\theta}\right)=p_{\Gamma(a_P,b_S)}\left(S_i-I_j\right)$ is the cumulative density of the sampling interval distribution, which is the probability of taking proposal path C.

Acceptance probability
The acceptance probability (removing the dependency on $\boldsymbol{\theta}$ after the first line, for readability) is

$$
\begin{aligned}
&\min\left[1,\frac{\Pr\left(\mathbf{S},\mathbf{G},\mathbf{Z}'\middle|\boldsymbol{\theta}\right)\times H\left(\mathbf{Z}\middle|\mathbf{Z}',\mathbf{S},\boldsymbol{\theta}\right)}{\Pr\left(\mathbf{S},\mathbf{G},\mathbf{Z}\middle|\boldsymbol{\theta}\right)\times H\left(\mathbf{Z}'\middle|\mathbf{Z},\mathbf{S},\boldsymbol{\theta}\right)}\right]\\
&=\min\left[1,\frac{\Pr\left(\mathbf{G}\middle|P'\right)\cdot\Pr\left(P'\middle|\mathbf{S},\mathbf{I}',\mathbf{M}'\right)\cdot\Pr\left(\mathbf{S}\middle|\mathbf{I}'\right)\cdot\Pr\left(\mathbf{I}',\mathbf{M}'\right)\times\Pr\left(P_i,P_j\middle|\mathbf{S},\mathbf{I},\mathbf{M}\right)\cdot 0.5\cdot p_{\Gamma(a_P,m_S)}\left(S_j-I_j\right)}{\Pr\left(\mathbf{G}\middle|P\right)\cdot\Pr\left(P\middle|\mathbf{S},\mathbf{I},\mathbf{M}\right)\cdot\Pr\left(\mathbf{S}\middle|\mathbf{I}\right)\cdot\Pr\left(\mathbf{I},\mathbf{M}\right)\times\Pr\left(P_i',P_j'\middle|\mathbf{S},\mathbf{I}',\mathbf{M}'\right)\cdot 0.5\cdot p_{\Gamma(a_P,m_S)}\left(S_i-I_j\right)}\right]\\
&=\min\left[1,\frac{\Pr\left(\mathbf{G}\middle|P'\right)\cdot\Pr\left(\mathbf{S}\middle|\mathbf{I}'\right)\cdot\Pr\left(\mathbf{I}',\mathbf{M}'\right)\cdot p_{\Gamma(a_P,m_S)}\left(S_j-I_j\right)}{\Pr\left(\mathbf{G}\middle|P\right)\cdot\Pr\left(\mathbf{S}\middle|\mathbf{I}\right)\cdot\Pr\left(\mathbf{I},\mathbf{M}\right)\cdot p_{\Gamma(a_P,m_S)}\left(S_i-I_j\right)}\right]
\end{aligned}
$$

Reversal
Reversal is possible through proposal path C, with focal host *j*. In Fig M1C, reversal takes place by proposing an infection time for host IV after its second transmission event. It is important to note that if host IV would not have a second transmission event, the reversal step is impossible; therefore, proposal path C is always rejected if the proposed new index ends up with only one secondary case.

Proposal path D {Proposal 1, Q123=NY}

Situation
Host *i* is not the index case, and I_i' is sampled before infection of the index case

Proposed changes to transmission tree

1. Topological changes:
    a. host *i* becomes index case, and the original index case *j* its first secondary case
2. Infection time change:
    a. I_i changes to I_i'

Proposal steps

2. host *i* becomes the index case: $M_i' = 0$
3. host *i* becomes the infector of the original index case: $M_j' = i$
4. bookkeeping: make corresponding changes in the nodes and move one of the coalescent nodes from host M_i to host *i*
5. propose new trees P_i' and P_{M_i}' by simulating the within-host coalescent model

Proposal distribution
The proposal distribution for I_i', M_i', M_j', P_i', and P_{M_i}' is

$$
\begin{aligned}
H\left(I_i', M_i', M_{M_i}', P_i', P_{M_i}' \middle| \mathbf{I}, \mathbf{M}, P, \mathbf{S}, \boldsymbol{\theta}\right)
&= \Pr\left(P_i', P_{M_i}' \middle| I_i', M_i', M_j', \mathbf{I}, \mathbf{M}, P, \mathbf{S}, \boldsymbol{\theta}\right) \cdot \\
&\quad \Pr\left(M_i', M_j' \middle| I_i', \mathbf{I}, \mathbf{M}, P, \mathbf{S}, \boldsymbol{\theta}\right) \cdot \\
&\quad \Pr\left(I_i' \middle| \mathbf{I}, \mathbf{M}, P, \mathbf{S}, \boldsymbol{\theta}\right) \\
&= \Pr\left(P_i' \middle| \mathbf{I'}, \mathbf{M'}, S_i, \boldsymbol{\theta}\right) \cdot \Pr\left(P_{M_i}' \middle| \mathbf{I'}, \mathbf{M'}, S_{M_i}, \boldsymbol{\theta}\right) \cdot \Pr\left(M_i', M_j' \middle| \mathbf{I'}, \boldsymbol{\theta}\right) \cdot \Pr\left(I_i' \middle| S_i\right) \\
&= \Pr\left(P_i', P_{M_i}' \middle| \mathbf{I'}, \mathbf{M'}, \mathbf{S}, \boldsymbol{\theta}\right) \cdot d_{\Gamma(a_P, m_S)}\left(S_i - I_i'\right)
\end{aligned}
$$

Here, $\Pr\left(M_i', M_j' \middle| \ldots\right) = 1$ because it follows automatically from the proposed I_i'.

Acceptance probability
The acceptance probability (removing the dependency on θ after the first line, for readability) is

$$
\begin{aligned}
&\min\left[1, \frac{\Pr\left(\mathbf{S}, \mathbf{G}, \mathbf{Z}' \middle| \boldsymbol{\theta}\right) \times H\left(\mathbf{Z} \middle| \mathbf{Z}', \mathbf{S}, \boldsymbol{\theta}\right)}{\Pr\left(\mathbf{S}, \mathbf{G}, \mathbf{Z} \middle| \boldsymbol{\theta}\right) \times H\left(\mathbf{Z}' \middle| \mathbf{Z}, \mathbf{S}, \boldsymbol{\theta}\right)}\right] \\
&= \min\left[1, \frac{\Pr\left(\mathbf{G} \middle| P'\right) \cdot \Pr\left(P' \middle| \mathbf{S}, \mathbf{I'}, \mathbf{M'}\right) \cdot \Pr\left(\mathbf{S} \middle| \mathbf{I'}\right) \cdot \Pr\left(\mathbf{I'}, \mathbf{M'}\right) \times \Pr\left(P_i, P_{M_i} \middle| \mathbf{S}, \mathbf{I}, \mathbf{M}\right) \cdot F_{M_i}\left(I_i\right) \cdot d_{\Gamma(a_P, m_S)}\left(S_i - I_i\right)}{\Pr\left(\mathbf{G} \middle| P\right) \cdot \Pr\left(P \middle| \mathbf{S}, \mathbf{I}, \mathbf{M}\right) \cdot \Pr\left(\mathbf{S} \middle| \mathbf{I}\right) \cdot \Pr\left(\mathbf{I}, \mathbf{M}\right) \times \Pr\left(P_i', P_{M_i}' \middle| \mathbf{S}, \mathbf{I'}, \mathbf{M'}\right) \cdot d_{\Gamma(a_P, m_S)}\left(S_i - I_i'\right)}\right] \\
&= \min\left[1, \frac{\Pr\left(\mathbf{G} \middle| P'\right) \cdot \Pr\left(S_i \middle| I_i'\right) \cdot \Pr\left(\mathbf{I'}, \mathbf{M'}\right) \cdot F_{M_i}\left(I_i\right) \cdot d_{\Gamma(a_P, m_S)}\left(S_i - I_i\right)}{\Pr\left(\mathbf{G} \middle| P\right) \cdot \Pr\left(S_i \middle| I_i\right) \cdot \Pr\left(\mathbf{I}, \mathbf{M}\right) \cdot d_{\Gamma(a_P, m_S)}\left(S_i - I_i'\right)}\right]
\end{aligned}
$$

Here, $F_{M_i}\left(I_i\right)$ is the probability of proposing the original infector in the reverse proposal (path B).

Reversal
Reversal is possible through proposal path B. Fig M1B shows the reverse proposal path B from the original path D in Fig M1D.

Proposal path E {Proposal 1, Q123=NNY; Proposal 4, Q1=Y}
The text below is specific for proposal 1 (within a subtree) [details specific for proposal 4 are placed between square brackets].

Situation
Host *i* is not the index case [history host], and I_i' is sampled after infection of the index case [history host], but before the first transmission node of host *i*, if there is any.

Proposed changes to transmission tree

1. Topological changes:
    a. host *i* gets a (possibly) new infector
2. Infection time changes:
    a. I_i changes to I_i'

Proposal steps

2. propose a new infector M_i' from the proposal distribution $F_j(I_i')$, which is the probability to select infector *j* at time I_i' in the outbreak. The proposal distribution is given by

$$F_j(t) = \frac{u(t-I_j)\cdot\left(d_{\Gamma(a_G,b_G)}(t-I_j)+1/d_{ij}\right)}{1+\sum_k\left[u(t-I_k)\cdot\left(d_{\Gamma(a_G,b_G)}(t-I_k)+1/d_{ij}\right)\right]},$$

   so the probability to select infector *j* is proportional to the density of the transmission rate by that host plus a weight $1/d_{ij}$, with d_{ij} equal to the sequence distance between isolates from hosts *i* and *j* (see list of definitions). This proposal distribution gives a sampling weight to possible infectors based on their infection time and related to the genetic distance between isolates. [For *j* = 0 or *k* = 0, use $I_0 = -\infty$ and d_{i0} = 1.]
3. bookkeeping: make corresponding changes in the nodes and move one of the coalescent nodes from host M_i to host M_i'
4. propose new trees P_i', P_{M_i}', and $P_{M_i'}'$ by simulating the within-host coalescent model

Proposal distribution

The proposal distribution for I_i', M_i', P_i', P_{M_i}', and $P_{M_i'}'$ is

$$\begin{aligned}
H&\left(I_i',M_i',P_i',P_{M_i}',P_{M_i'}'\middle|\mathbf{I},\mathbf{M},P,\mathbf{S},\boldsymbol{\theta}\right)\\
&=\Pr\left(P_i',P_{M_i}',P_{M_i'}'\middle|I_i',M_i',\mathbf{I},\mathbf{M},P,\mathbf{S},\boldsymbol{\theta}\right)\cdot\\
&\quad\Pr\left(M_i'\middle|I_i',\mathbf{I},\mathbf{M},P,\mathbf{S},\boldsymbol{\theta}\right)\cdot\\
&\quad\Pr\left(I_i'\middle|\mathbf{I},\mathbf{M},P,\mathbf{S},\boldsymbol{\theta}\right)\\
&=\Pr\left(P_i'\middle|\mathbf{I'},\mathbf{M'},S_i,\boldsymbol{\theta}\right)\cdot\Pr\left(P_{M_i}'\middle|\mathbf{I'},\mathbf{M'},S_{M_i},\boldsymbol{\theta}\right)\cdot\Pr\left(P_{M_i'}'\middle|\mathbf{I'},\mathbf{M'},S_{M_i'},\boldsymbol{\theta}\right)\cdot\\
&\quad\Pr\left(M_i'\middle|\mathbf{I'},\boldsymbol{\theta}\right)\cdot\Pr\left(I_i'\middle|S_i,\boldsymbol{\theta}\right)\\
&=\Pr\left(P_i',P_{M_i}',P_{M_i'}'\middle|\mathbf{I'},\mathbf{M'},\mathbf{S},\boldsymbol{\theta}\right)\cdot F_{M_i'}(I_i')\cdot d_{\Gamma(a_P,m_S)}(S_i-I_i')
\end{aligned}$$

Acceptance probability

The acceptance probability (removing the dependency on θ after the first line, for readability) is

$$\min\left[1,\frac{\Pr(\mathbf{S},\mathbf{G},\mathbf{Z}'|\boldsymbol{\theta})\times H(\mathbf{Z}|\mathbf{Z}',\mathbf{S},\boldsymbol{\theta})}{\Pr(\mathbf{S},\mathbf{G},\mathbf{Z}|\boldsymbol{\theta})\times H(\mathbf{Z}'|\mathbf{Z},\mathbf{S},\boldsymbol{\theta})}\right]$$

$$=\min\left[1,\frac{\Pr(\mathbf{G}|P')\cdot\Pr(P'|\mathbf{S},\mathbf{I}',\mathbf{M}')\cdot\Pr(\mathbf{S}|\mathbf{I}')\cdot\Pr(\mathbf{I}',\mathbf{M}')\times\Pr(P_i,P_{M_i},P_{M_i'}|\mathbf{S},\mathbf{I},\mathbf{M})\cdot F_{M_i}(I_i)\cdot d_{\Gamma(a_P,m_S)}(S_i-I_i)}{\Pr(\mathbf{G}|P)\cdot\Pr(P|\mathbf{S},\mathbf{I},\mathbf{M})\cdot\Pr(\mathbf{S}|\mathbf{I})\cdot\Pr(\mathbf{I},\mathbf{M})\times\Pr(P_i',P_{M_i}',P_{M_i'}'|\mathbf{S},\mathbf{I}',\mathbf{M}')\cdot F_{M_i'}(I_i')\cdot d_{\Gamma(a_P,m_S)}(S_i-I_i')}\right]$$

$$=\min\left[1,\frac{\Pr(\mathbf{G}|P')\cdot\Pr(S_i|I_i')\cdot\Pr(\mathbf{I}',\mathbf{M}')\cdot F_{M_i}(I_i)\cdot d_{\Gamma(a_P,m_S)}(S_i-I_i)}{\Pr(\mathbf{G}|P)\cdot\Pr(S_i|I_i)\cdot\Pr(\mathbf{I},\mathbf{M})\cdot F_{M_i'}(I_i')\cdot d_{\Gamma(a_P,m_S)}(S_i-I_i')}\right]$$

Reversal
Reversal is possible through proposal path E, with the original infection time I_i proposed for the same focal host *i* (going back in Fig M1E).

Proposal path F {Proposal 1, Q12=NNY}

Situation
Host *i* is not the index case and I_i' is sampled after infection of its first infectee *j*

Proposed changes to transmission tree

1. Topological changes:
    a. M_i becomes infector of host *j*
    b. host *j* becomes infector of host *i*
    c. with 50% probability: the other secondary cases of hosts *i* and *j* are exchanged
2. Infection time change:
    a. the sampled I_i' is discarded; instead, the infection times of hosts *i* and *j* are switched

Proposal steps

2. discard the sampled infection time and switch infection times by proposing $I_i'=I_j$ and $I_j'=I_i$
3. switch role by proposing $M_i'=j$ and $M_j'=M_i$
4. bookkeeping: make corresponding changes in the nodes and move one of the coalescent nodes from host *i* to host *j*
5. with 50% probability: for all $\{k|M_k=i\wedge k\neq j\}$, propose $M_k'=j$; for all $\{k|M_k=j\}$, propose $M_k'=i$; finish by bookkeeping: swap all transmission and coalescent nodes between P_i and P_j
6. propose new trees P_i' and P_j' by simulating the within-host coalescent model

Proposal distribution
The proposal distribution for I_i', I_j', M_i', M_j', P_i', and P_j' is

$$
\begin{aligned}
&H\left(I_i',I_j',\mathbf{M}',M_j',P_i',P_j'\middle|\mathbf{I},\mathbf{M},P,\mathbf{S},\boldsymbol{\theta}\right)\\
&\qquad=\Pr\left(P_i',P_j'\middle|I_i',I_j',M_i',M_j',\mathbf{I},\mathbf{M},P,\mathbf{S},\boldsymbol{\theta}\right)\cdot\\
&\qquad\quad\Pr\left(\mathbf{M}'\middle|I_i',I_j',\mathbf{I},\mathbf{M},P,\mathbf{S},\boldsymbol{\theta}\right)\cdot\\
&\qquad\quad\Pr\left(I_i',I_j'\middle|\mathbf{I},\mathbf{M},P,\mathbf{S},\boldsymbol{\theta}\right)\\
&\qquad=\Pr\left(P_i'\middle|\mathbf{I}',\mathbf{M}',S_i,\boldsymbol{\theta}\right)\cdot\Pr\left(P_j'\middle|\mathbf{I}',\mathbf{M}',S_j,\boldsymbol{\theta}\right)\cdot\\
&\qquad\quad\Pr\left(\mathbf{M}'\middle|\mathbf{I}',\boldsymbol{\theta}\right)\cdot\Pr\left(I_j'\middle|I_i',\boldsymbol{\theta}\right)\cdot\Pr\left(I_i'\middle|\mathbf{I},S_i,\boldsymbol{\theta}\right)\\
&\qquad=\Pr\left(P_i',P_j'\middle|\mathbf{I}',\mathbf{M}',\mathbf{S},\boldsymbol{\theta}\right)\cdot 0.5\cdot p_{\Gamma(a_P,m_S)}\left(S_i-I_j\right)
\end{aligned}
$$

Here, $\Pr\left(\mathbf{M}'\middle|\mathbf{I}',\boldsymbol{\theta}\right)=0.5$ because of the two possible rearrangements of infectees that follow automatically from the proposed I_i', and $\Pr\left(I_j'\middle|I_i',\boldsymbol{\theta}\right)=1$ because it follows automatically from the proposed I_i'. In the last step, $\Pr\left(I_i'\middle|\mathbf{I},S_i,\boldsymbol{\theta}\right)=p_{\Gamma(a_P,b_S)}\left(S_i-I_j\right)$ is the cumulative density of the sampling interval distribution, which is the probability of taking proposal path F.

Acceptance probability

The acceptance probability (removing the dependency on $\boldsymbol{\theta}$ after the first line, for readability) is

$$
\begin{aligned}
&\min\left[1,\frac{\Pr\left(\mathbf{S},\mathbf{G},\mathbf{Z}'\middle|\boldsymbol{\theta}\right)\times H\left(\mathbf{Z}\middle|\mathbf{Z}',\mathbf{S},\boldsymbol{\theta}\right)}{\Pr\left(\mathbf{S},\mathbf{G},\mathbf{Z}\middle|\boldsymbol{\theta}\right)\times H\left(\mathbf{Z}'\middle|\mathbf{Z},\mathbf{S},\boldsymbol{\theta}\right)}\right]\\
&=\min\left[1,\frac{\Pr\left(\mathbf{G}\middle|P'\right)\cdot\Pr\left(P'\middle|\mathbf{S},\mathbf{I}',\mathbf{M}'\right)\cdot\Pr\left(\mathbf{S}\middle|\mathbf{I}'\right)\cdot\Pr\left(\mathbf{I}',\mathbf{M}'\right)\times\Pr\left(P_i,P_j\middle|\mathbf{S},\mathbf{I},\mathbf{M}\right)\cdot 0.5\cdot p_{\Gamma(a_P,m_S)}\left(S_j-I_j\right)}{\Pr\left(\mathbf{G}\middle|P\right)\cdot\Pr\left(P\middle|\mathbf{S},\mathbf{I},\mathbf{M}\right)\cdot\Pr\left(\mathbf{S}\middle|\mathbf{I}\right)\cdot\Pr\left(\mathbf{I},\mathbf{M}\right)\times\Pr\left(P_i',P_j'\middle|\mathbf{S},\mathbf{I}',\mathbf{M}'\right)\cdot 0.5\cdot p_{\Gamma(a_P,m_S)}\left(S_i-I_j\right)}\right]\\
&=\min\left[1,\frac{\Pr\left(\mathbf{G}\middle|P'\right)\cdot\Pr\left(\mathbf{S}\middle|\mathbf{I}'\right)\cdot\Pr\left(\mathbf{I}',\mathbf{M}'\right)\cdot p_{\Gamma(a_P,m_S)}\left(S_j-I_j\right)}{\Pr\left(\mathbf{G}\middle|P\right)\cdot\Pr\left(\mathbf{S}\middle|\mathbf{I}\right)\cdot\Pr\left(\mathbf{I},\mathbf{M}\right)\cdot p_{\Gamma(a_P,m_S)}\left(S_i-I_j\right)}\right]
\end{aligned}
$$

Reversal

Reversal is possible through proposal path F, with focal host *j*. In Fig M1F, reversal takes place by proposing an infection time for host III after the transmission time to host IV.

Proposal path G {Proposal 2, Q12=YY}

Situation

Host *i* is the index case, and I_i' is before its first coalescent node.

Proposed changes to transmission tree

1. Topological changes: none
2. Infection time changes:
    a. I_i changes to I_i'

Proposal steps

2. bookkeeping: propose P_i' by adjusting the infection time

Proposal distribution

The proposal distribution for I_i' and P_i' is

$$H(I_i',P_i'|\mathbf{I},\mathbf{M},P,\mathbf{S},\boldsymbol{\theta}) = \Pr(P_i'|I_i',\mathbf{I},\mathbf{M},P,\mathbf{S},\boldsymbol{\theta}) \cdot \Pr(I_i'|\mathbf{I},\mathbf{M},P,\mathbf{S},\boldsymbol{\theta}) = \Pr(I_i'|S_i) = d_{\Gamma(a_P,m_S)}(S_i - I_i')$$

Here, $\Pr(P_i'|...) = 1$, because it follows automatically from the proposed I_i'.

Acceptance probability

The acceptance probability (removing the dependency on θ after the first line, for readability) is

$$\min\left[1, \frac{\Pr(\mathbf{S},\mathbf{G},\mathbf{Z}'|\boldsymbol{\theta}) \times H(\mathbf{Z}|\mathbf{Z}',\mathbf{S},\boldsymbol{\theta})}{\Pr(\mathbf{S},\mathbf{G},\mathbf{Z}|\boldsymbol{\theta}) \times H(\mathbf{Z}'|\mathbf{Z},\mathbf{S},\boldsymbol{\theta})}\right]$$

$$= \min\left[1, \frac{\Pr(\mathbf{G}|P') \cdot \Pr(P'|\mathbf{S},\mathbf{I}',\mathbf{M}) \cdot \Pr(\mathbf{S}|\mathbf{I}') \cdot \Pr(\mathbf{I}',\mathbf{M}) \times d_{\Gamma(a_P,m_S)}(S_i - I_i)}{\Pr(\mathbf{G}|P) \cdot \Pr(P|\mathbf{S},\mathbf{I},\mathbf{M}) \cdot \Pr(\mathbf{S}|\mathbf{I}) \cdot \Pr(\mathbf{I},\mathbf{M}) \times d_{\Gamma(a_P,m_S)}(S_i - I_i')}\right]$$

$$= \min\left[1, \frac{\Pr(P_i'|S_i,\mathbf{I}',\mathbf{M}) \cdot \Pr(S_i|I_i') \cdot \Pr(\mathbf{I}',\mathbf{M}) \cdot d_{\Gamma(a_P,m_S)}(S_i - I_i)}{\Pr(P_i|S_i,\mathbf{I},\mathbf{M}) \cdot \Pr(S_i|I_i) \cdot \Pr(\mathbf{I},\mathbf{M}) \cdot d_{\Gamma(a_P,m_S)}(S_i - I_i')}\right]$$

Here, $\Pr(\mathbf{G}|P') = \Pr(\mathbf{G}|P)$, because it does not depend on the infection time of the index case.

Reverse proposal

This sampling step can be reversed by proposing through (the same) proposal path G, with the original infection time I_i proposed for the same focal host *i* (similar to path A, Fig M1A).

Proposal path H {Proposal 2, Q12=NY}

Situation

Host *i* is not the index case, and I_i' is after the MRCA of the sampling nodes in hosts *i* and M_i.

Proposed changes to transmission tree

1. Topological changes:
    a. transmission nodes move between host *i* and its infector, if I_i and I_i' are on different branches in the phylogenetic tree *P*
2. Infection time changes:
    a. I_i changes to I_i'

Proposal steps

2. bookkeeping: change h_x for all nodes *x* involved: if I_i and I_i' are on different branches in the phylogenetic tree *P*, coalescent nodes and transmission nodes move from host *i* to host M_i if $I_i' > I_i$, and vice versa if $I_i' < I_i$.

Proposal distribution

The proposal distribution for I_i', P_i', and P_{M_i}' is

$$
\begin{aligned}
H\left(I_i',\mathbf{M}',P_i',P_{M_i}'\middle|\mathbf{I},\mathbf{M},P,\mathbf{S},\boldsymbol{\theta}\right)
&=\Pr\left(P_i',P_{M_i}'\middle|I_i',\mathbf{M}',\mathbf{I},\mathbf{M},P,\mathbf{S},\boldsymbol{\theta}\right)\cdot\\
&\quad\Pr\left(\mathbf{M}'\middle|I_i',\mathbf{I},\mathbf{M},P,\mathbf{S},\boldsymbol{\theta}\right)\cdot\\
&\quad\Pr\left(I_i'\middle|\mathbf{I},\mathbf{M},P,\mathbf{S},\boldsymbol{\theta}\right)\\
&=d_{\Gamma(a_P,m_S)}\left(S_i-I_i'\right)
\end{aligned}
$$

Here, $\Pr\left(P_i',P_{M_i}'\middle|...\right)=1$ and $\Pr\left(\mathbf{M}'\middle|...\right)=1$ because these follow automatically from the proposed I_i'.

Acceptance probability

The acceptance probability (removing the dependency on $\boldsymbol{\theta}$ after the first line, for readability) is

$$
\begin{aligned}
&\min\left[1,\frac{\Pr\left(\mathbf{S},\mathbf{G},\mathbf{Z}'\middle|\boldsymbol{\theta}\right)\times H\left(\mathbf{Z}\middle|\mathbf{Z}',\mathbf{S},\boldsymbol{\theta}\right)}{\Pr\left(\mathbf{S},\mathbf{G},\mathbf{Z}\middle|\boldsymbol{\theta}\right)\times H\left(\mathbf{Z}'\middle|\mathbf{Z},\mathbf{S},\boldsymbol{\theta}\right)}\right]\\
&=\min\left[1,\frac{\Pr\left(\mathbf{G}\middle|P'\right)\cdot\Pr\left(P'\middle|\mathbf{S},\mathbf{I}',\mathbf{M}'\right)\cdot\Pr\left(\mathbf{S}\middle|\mathbf{I}'\right)\cdot\Pr\left(\mathbf{I}',\mathbf{M}'\right)\times d_{\Gamma(a_P,m_S)}\left(S_i-I_i\right)}{\Pr\left(\mathbf{G}\middle|P\right)\cdot\Pr\left(P\middle|\mathbf{S},\mathbf{I},\mathbf{M}\right)\cdot\Pr\left(\mathbf{S}\middle|\mathbf{I}\right)\cdot\Pr\left(\mathbf{I},\mathbf{M}\right)\times d_{\Gamma(a_P,m_S)}\left(S_i-I_i'\right)}\right]\\
&=\min\left[1,\frac{\Pr\left(P_i',P_{M_i}'\middle|S_i,\mathbf{I}',\mathbf{M}'\right)\cdot\Pr\left(S_i\middle|I_i'\right)\cdot\Pr\left(\mathbf{I}',\mathbf{M}'\right)\cdot d_{\Gamma(a_P,m_S)}\left(S_i-I_i\right)}{\Pr\left(P_i,P_{M_i}\middle|S_i,\mathbf{I},\mathbf{M}\right)\cdot\Pr\left(S_i\middle|I_i\right)\cdot\Pr\left(\mathbf{I},\mathbf{M}\right)\cdot d_{\Gamma(a_P,m_S)}\left(S_i-I_i'\right)}\right]
\end{aligned}
$$

Here, $\Pr\left(\mathbf{G}\middle|P'\right)=\Pr\left(\mathbf{G}\middle|P\right)$, because the phylogenetic tree as a whole does not change.

Reverse proposal

This sampling step can be reversed by proposing through (the same) proposal path H, with the original infection time I_i proposed for the same focal host *i* (going back in Fig M2A).

Proposal path I {Proposal 2, Q1234=NNYY}

Situation

Host M_i is the index case, I_i' is before the MRCA of the sampling nodes in hosts *i* and M_i, and I_{M_i}' is after the MRCA of the sampling nodes *i* and M_i.

Proposed changes to transmission tree

1. Topological changes:
    a. host *i* becomes the index case, and host M_i its secondary case
    b. transmission nodes move from host M_i to host *i*, consistent with the branch on which I_{M_i}' is placed
2. Infection time changes:
    a. I_i changes to I_{M_i}
    b. I_{M_i} changes to I_{M_i}'

Proposal steps (after steps 1 and 2 to propose I_i' and I_{M_i}')

3. discard the proposed I_i'; instead, propose $I_i'=I_{M_i}$

4. host *i* becomes the index case: $M_i' = 0$
5. host M_i gets host *i* as infector: $M_{M_i}' = i$
6. bookkeeping: propose other new infectors $\mathbf{M}'$ by changing h_x for all nodes *x* involved: some coalescent nodes and transmission nodes move from host M_i to host *i*.

Proposal distribution

The proposal distribution for I_i', I_{M_i}', $\mathbf{M}'$, P_i', and P_{M_i}' is

$$\begin{aligned} H\left(I_i',I_{M_i}',\mathbf{M}',P_i',P_{M_i}'\middle|\mathbf{I},\mathbf{M},P,\mathbf{S},\boldsymbol{\theta}\right) \\ &= \Pr\left(P_i',P_{M_i}'\middle|I_i',I_{M_i}',\mathbf{M}',\mathbf{I},\mathbf{M},P,\mathbf{S},\boldsymbol{\theta}\right)\cdot \\ &\quad \Pr\left(\mathbf{M}'\middle|I_i',I_{M_i}',\mathbf{I},\mathbf{M},P,\mathbf{S},\boldsymbol{\theta}\right)\cdot \\ &\quad \Pr\left(I_i',I_{M_i}'\middle|\mathbf{I},\mathbf{M},P,\mathbf{S},\boldsymbol{\theta}\right) \\ &= \left(1-p_{\Gamma(a_P,m_S)}\left(S_i-t_{mrca(i,M_i)}\right)\right)\cdot d_{\Gamma(a_P,m_S)}\left(S_{M_i}-I_{M_i}'\right) \end{aligned}$$

Here, $\Pr\left(P_i',P_{M_i}'\middle|\ldots\right)=1$ and $\Pr\left(\mathbf{M}'\middle|\ldots\right)=1$ because these follow automatically from the proposed I_i' and I_{M_i}'. In the last step, $1-p_{\Gamma(a_P,m_S)}\left(S_i-t_{mrca(i,M_i)}\right)$ is 1 minus the cumulative density of the proposal distribution, which is the probability of taking proposal path I (conditional on $I_{M_i}' > t_{mrca(i,M_i)}$).

Acceptance probability

The acceptance probability (removing the dependency on $\boldsymbol{\theta}$ after the first line, for readability) is

$$\begin{aligned} &\min\left[1,\frac{\Pr\left(\mathbf{S},\mathbf{G},\mathbf{Z}'\middle|\boldsymbol{\theta}\right)\times H\left(\mathbf{Z}\middle|\mathbf{Z}',\mathbf{S},\boldsymbol{\theta}\right)}{\Pr\left(\mathbf{S},\mathbf{G},\mathbf{Z}\middle|\boldsymbol{\theta}\right)\times H\left(\mathbf{Z}'\middle|\mathbf{Z},\mathbf{S},\boldsymbol{\theta}\right)}\right] \\ &=\min\left[1,\frac{\Pr\left(\mathbf{G}\middle|P'\right)\cdot\Pr\left(P'\middle|\mathbf{S},\mathbf{I}',\mathbf{M}'\right)\cdot\Pr\left(\mathbf{S}\middle|\mathbf{I}'\right)\cdot\Pr\left(\mathbf{I}',\mathbf{M}'\right)\times\left(1-p_{\Gamma(a_P,m_S)}\left(S_{M_i}-t_{mrca(i,M_i)}\right)\right)\cdot d_{\Gamma(a_P,m_S)}\left(S_i-I_i\right)}{\Pr\left(\mathbf{G}\middle|P\right)\cdot\Pr\left(P\middle|\mathbf{S},\mathbf{I},\mathbf{M}\right)\cdot\Pr\left(\mathbf{S}\middle|\mathbf{I}\right)\cdot\Pr\left(\mathbf{I},\mathbf{M}\right)\times\left(1-p_{\Gamma(a_P,m_S)}\left(S_i-t_{mrca(i,M_i)}\right)\right)\cdot d_{\Gamma(a_P,m_S)}\left(S_{M_i}-I_{M_i}'\right)}\right] \\ &=\min\left[1,\frac{\Pr\left(P_i',P_{M_i}'\middle|S_i,S_{M_i},\mathbf{I}',\mathbf{M}'\right)\cdot\Pr\left(S_i,S_{M_i}\middle|I_i',I_{M_i}'\right)\cdot\Pr\left(\mathbf{I}',\mathbf{M}'\right)\cdot\left(1-p_{\Gamma(.)}\left(S_{M_i}-t_{mrca(i,M_i)}\right)\right)\cdot d_{\Gamma(.)}\left(S_i-I_i\right)}{\Pr\left(P_i,P_{M_i}\middle|S_i,S_{M_i},\mathbf{I},\mathbf{M}\right)\cdot\Pr\left(S_i,S_{M_i}\middle|I_i,I_{M_i}\right)\cdot\Pr\left(\mathbf{I},\mathbf{M}\right)\cdot\left(1-p_{\Gamma(.)}\left(S_i-t_{mrca(i,M_i)}\right)\right)\cdot d_{\Gamma(.)}\left(S_{M_i}-I_{M_i}'\right)}\right] \end{aligned}$$

Reverse proposal

This sampling step can be reversed by proposing through (the same) proposal path I, with the original index case (now secondary case) as focal host. In Fig M2B, reversal occurs by first proposing any infection time before $\mathrm{MRCA}_{\mathrm{I,II}}$ for host I, and then proposing for host II its original infection time.

Proposal path J {Proposal 2, Q12345=NNYNY}

Situation

Hosts *i* and M_i are not the index case, I_i' is before the MRCA of the sampling nodes in hosts *i* and M_i but after the MRCA of the sampling nodes in hosts *i* and M_{M_i}, and I_{M_i}' is after the MRCA of the sampling nodes in hosts *i* and M_i.

Proposed changes to transmission tree

1. Topological changes:
    a. host M_{M_i} becomes the infector of host *i*
    b. host *i* becomes the infector of host M_i
    c. transmission nodes move from hosts M_i and M_{M_i} to host *i*, consistent with the branches on which I_i' and I_{M_i}' are placed
2. Infection time changes:
    a. I_i changes to I_i'
    b. I_{M_i} changes to I_{M_i}'

Proposal steps (after steps 1 and 2 to propose I_i' and I_{M_i}')

3. switch role in transmission tree by proposing $M_i' = M_{M_i}$ and $M_{M_i}' = i$
4. bookkeeping: propose other new infectors $\mathbf{M}'$ by changing h_x for all nodes *x* involved: some coalescent nodes and transmission nodes move from hosts M_i and M_{M_i} to host *i*.

Proposal distribution
The proposal distribution for I_i', I_{M_i}', P_i', and P_{M_i}' is

$$\begin{aligned}
&H\left(I_i', I_{M_i}', \mathbf{M}', P_i', P_{M_i}' \middle| \mathbf{I}, \mathbf{M}, P, \mathbf{S}, \boldsymbol{\theta}\right) \\
&= \Pr\left(P_i', P_{M_i}' \middle| I_i', I_{M_i}', \mathbf{M}', \mathbf{I}, \mathbf{M}, P, \mathbf{S}, \boldsymbol{\theta}\right) \cdot \\
&\quad \Pr\left(\mathbf{M}' \middle| I_i', I_{M_i}', \mathbf{I}, \mathbf{M}, P, \mathbf{S}, \boldsymbol{\theta}\right) \cdot \\
&\quad \Pr\left(I_i', I_{M_i}' \middle| \mathbf{I}, \mathbf{M}, P, \mathbf{S}, \boldsymbol{\theta}\right) \\
&= d_{\Gamma(a_P, m_S)}\left(S_i - I_i'\right) \cdot d_{\Gamma(a_P, m_S)}\left(S_{M_i} - I_{M_i}'\right)
\end{aligned}$$

Here, $\Pr\left(P_i', P_{M_i}' \middle| \ldots\right) = 1$ and $\Pr\left(\mathbf{M}' \middle| \ldots\right) = 1$ because these follow automatically from the proposed I_i' and I_{M_i}'.

Acceptance probability
The acceptance probability (removing the dependency on θ after the first line, for readability) is

$$\begin{aligned}
&\min\left[1, \frac{\Pr\left(\mathbf{S}, \mathbf{G}, \mathbf{Z}' \middle| \boldsymbol{\theta}\right) \times H\left(\mathbf{Z} \middle| \mathbf{Z}', \mathbf{S}, \boldsymbol{\theta}\right)}{\Pr\left(\mathbf{S}, \mathbf{G}, \mathbf{Z} \middle| \boldsymbol{\theta}\right) \times H\left(\mathbf{Z}' \middle| \mathbf{Z}, \mathbf{S}, \boldsymbol{\theta}\right)}\right] \\
&= \min\left[1, \frac{\Pr\left(\mathbf{G} \middle| P'\right) \cdot \Pr\left(P' \middle| \mathbf{S}, \mathbf{I}', \mathbf{M}'\right) \cdot \Pr\left(\mathbf{S} \middle| \mathbf{I}'\right) \cdot \Pr\left(\mathbf{I}', \mathbf{M}'\right) \times d_{\Gamma(a_P, m_S)}\left(S_i - I_i\right) \cdot d_{\Gamma(a_P, m_S)}\left(S_{M_i} - I_{M_i}\right)}{\Pr\left(\mathbf{G} \middle| P\right) \cdot \Pr\left(P \middle| \mathbf{S}, \mathbf{I}, \mathbf{M}\right) \cdot \Pr\left(\mathbf{S} \middle| \mathbf{I}\right) \cdot \Pr\left(\mathbf{I}, \mathbf{M}\right) \times d_{\Gamma(a_P, m_S)}\left(S_i - I_i'\right) \cdot d_{\Gamma(a_P, m_S)}\left(S_{M_i} - I_{M_i}'\right)}\right] \\
&= \min\left[1, \frac{\Pr\left(P_i', P_{M_i}' \middle| S_i, S_{M_i}, \mathbf{I}', \mathbf{M}'\right) \cdot \Pr\left(S_i, S_{M_i} \middle| I_i', I_{M_i}'\right) \cdot \Pr\left(\mathbf{I}', \mathbf{M}'\right) \cdot d_{\Gamma(a_P, m_S)}\left(S_i - I_i\right) \cdot d_{\Gamma(a_P, m_S)}\left(S_{M_i} - I_{M_i}\right)}{\Pr\left(P_i, P_{M_i} \middle| S_i, S_{M_i}, \mathbf{I}, \mathbf{M}\right) \cdot \Pr\left(S_i, S_{M_i} \middle| I_i, I_{M_i}\right) \cdot \Pr\left(\mathbf{I}, \mathbf{M}\right) \cdot d_{\Gamma(a_P, m_S)}\left(S_i - I_i'\right) \cdot d_{\Gamma(a_P, m_S)}\left(S_{M_i} - I_{M_i}'\right)}\right]
\end{aligned}$$

Reverse proposal
This sampling step can be reversed by proposing through (the same) proposal path J, with the original infector (now secondary case) as focal host. In Fig M2C, reversal occurs by first proposing the original infection time for host II (which is between the two MRCAs), and then proposing the original infection time for host III.

Proposal path K {Proposal 3}

Situation
Any.

Proposed changes to transmission tree
None.

Proposal steps

2. discard the sampled infection time I_i'
3. propose new tree P_i' by simulating only new $v_{x|x\in P_i}$ (a new topology, not coalescent times)

Proposal distribution
The proposal distribution for P_i' is

$$H\left(P_i'\middle|\mathbf{I},\mathbf{M},P,\mathbf{S},\boldsymbol{\theta}\right)=\Pr\left(P_i'\middle|\mathbf{I},\mathbf{M},S_i,\boldsymbol{\theta}\right)$$

Acceptance probability
The acceptance probability (removing the dependency on θ after the first line, for readability) is

$$\begin{aligned}&\min\left[1,\frac{\Pr\left(\mathbf{S},\mathbf{G},\mathbf{Z}'\middle|\boldsymbol{\theta}\right)\times H\left(\mathbf{Z}\middle|\mathbf{Z}',\mathbf{S},\boldsymbol{\theta}\right)}{\Pr\left(\mathbf{S},\mathbf{G},\mathbf{Z}\middle|\boldsymbol{\theta}\right)\times H\left(\mathbf{Z}'\middle|\mathbf{Z},\mathbf{S},\boldsymbol{\theta}\right)}\right]\\&=\min\left[1,\frac{\Pr\left(\mathbf{G}\middle|P'\right)\cdot\Pr\left(P'\middle|\mathbf{S},\mathbf{I},\mathbf{M}\right)\cdot\Pr\left(\mathbf{S}\middle|\mathbf{I}\right)\cdot\Pr\left(\mathbf{I},\mathbf{M}\right)\times\Pr\left(P_i\middle|\mathbf{S},\mathbf{I},\mathbf{M}\right)}{\Pr\left(\mathbf{G}\middle|P\right)\cdot\Pr\left(P\middle|\mathbf{S},\mathbf{I},\mathbf{M}\right)\cdot\Pr\left(\mathbf{S}\middle|\mathbf{I}\right)\cdot\Pr\left(\mathbf{I},\mathbf{M}\right)\times\Pr\left(P_i'\middle|\mathbf{S},\mathbf{I},\mathbf{M}\right)}\right]\\&=\min\left[1,\frac{\Pr\left(\mathbf{G}\middle|P'\right)}{\Pr\left(\mathbf{G}\middle|P\right)}\right]\end{aligned}$$

Reversal
Reversal is possible through the same proposal path K, by resampling the original P_i.

Proposal path L {Proposal 5}

Situation
Any.

Proposed changes to transmission tree
None.

Proposal steps

1. propose new tree P_0' by simulating only new $t_{x|x\in P_0}$ (new coalescent times only)

Proposal distribution
The proposal distribution for P_0' is

$$H\left(P_0'\middle|\mathbf{I},\mathbf{M},P,\mathbf{S},\boldsymbol{\theta}\right)=\Pr\left(P_0'\middle|\mathbf{I},\mathbf{M},\boldsymbol{\theta}\right)$$

Acceptance probability
The acceptance probability (removing the dependency on θ after the first line, for readability) is

$$
\begin{aligned}
&\min\left[1, \frac{\Pr\left(\mathbf{S},\mathbf{G},\mathbf{Z}'\middle|\boldsymbol{\theta}\right)\times H\left(\mathbf{Z}\middle|\mathbf{Z}',\mathbf{S},\boldsymbol{\theta}\right)}{\Pr\left(\mathbf{S},\mathbf{G},\mathbf{Z}\middle|\boldsymbol{\theta}\right)\times H\left(\mathbf{Z}'\middle|\mathbf{Z},\mathbf{S},\boldsymbol{\theta}\right)}\right] \\
&= \min\left[1, \frac{\Pr\left(\mathbf{G}\middle|P'\right)\cdot\Pr\left(P'\middle|\mathbf{S},\mathbf{I},\mathbf{M}\right)\cdot\Pr\left(\mathbf{S}\middle|\mathbf{I}\right)\cdot\Pr\left(\mathbf{I},\mathbf{M}\right)\times\Pr\left(P_i\middle|\mathbf{S},\mathbf{I},\mathbf{M}\right)}{\Pr\left(\mathbf{G}\middle|P\right)\cdot\Pr\left(P\middle|\mathbf{S},\mathbf{I},\mathbf{M}\right)\cdot\Pr\left(\mathbf{S}\middle|\mathbf{I}\right)\cdot\Pr\left(\mathbf{I},\mathbf{M}\right)\times\Pr\left(P_i'\middle|\mathbf{S},\mathbf{I},\mathbf{M}\right)}\right] \\
&= \min\left[1, \frac{\Pr\left(\mathbf{G}\middle|P'\right)}{\Pr\left(\mathbf{G}\middle|P\right)}\right]
\end{aligned}
$$

Reversal
Reversal is possible through the same proposal path L, by resampling the original P_0.

Irreducibility of the MCMC chain

Here we argue heuristically that the MCMC chain is irreducible, i.e. any configuration of the transmission tree and phylogenetic tree consistent with the sampling times can be reached from any (current) configuration:

- for every host *i*, it is possible to reach any infection time I_i (prior to its sampling time S_i), without changing the other hosts' infection times:
    - if host *i* does currently not have secondary cases:
        - sample any infection time with host *i* as focal host, followed by proposal path D or E
    - if host *i* does currently have secondary cases, but is not the index case:
        - first, lose all secondary cases of host *i* by taking these secondary cases as focal hosts, sampling the infection times they already have, thus following proposal path E, and proposing alternative infectors
        - then, sample any infection time with host *i* as focal host, followed by proposal path D or E
    - if host *i* is currently the index case:
        - first, lose the index case status by a single proposal path B with host *i* as focal host
        - then, lose all secondary cases of host *i* by taking these secondary cases as focal hosts, sampling the infection times they already have, thus following proposal path E, and proposing alternative infectors
        - then, sample any infection time with host *i* as focal host, followed by proposal path D or E
- for every set of infection times **I**, all transmission trees consistent with those times can be reached:
    - with host *i* as focal host, sample the infection time it already has, and follow proposal path A (if host *i* is index case) or proposal path E (otherwise), and propose any host infected before I_i as infector.
- for every transmission tree, all phylogenetic trees consistent with that tree can be reached:
    - with host *i* as focal host, sample the infection time it already has, follow proposal path A (if host *i* is index case) or proposal path E (otherwise), sample the infector it already has, and simulate the phylogenetic minitree in host *i* (and its infector).
    - with host $i = 0$ as focal host, follow proposal path L for coalescent times given a topology. Each topology can be reached from sampling index cases with proposal paths A, and reconnecting them to the phylogenetic tree in the history host.

References

1. Felsenstein J. Evolutionary trees from DNA sequences: a maximum likelihood approach. J Mol Evol. 1981;17(6):368-76. PubMed PMID: 7288891.
2. Roberts GO, Gelman A, Gilks WR. Weak convergence and optimal scaling of random walk metropolis algorithms. Ann Appl Prob. 1997;7(1):110-20.

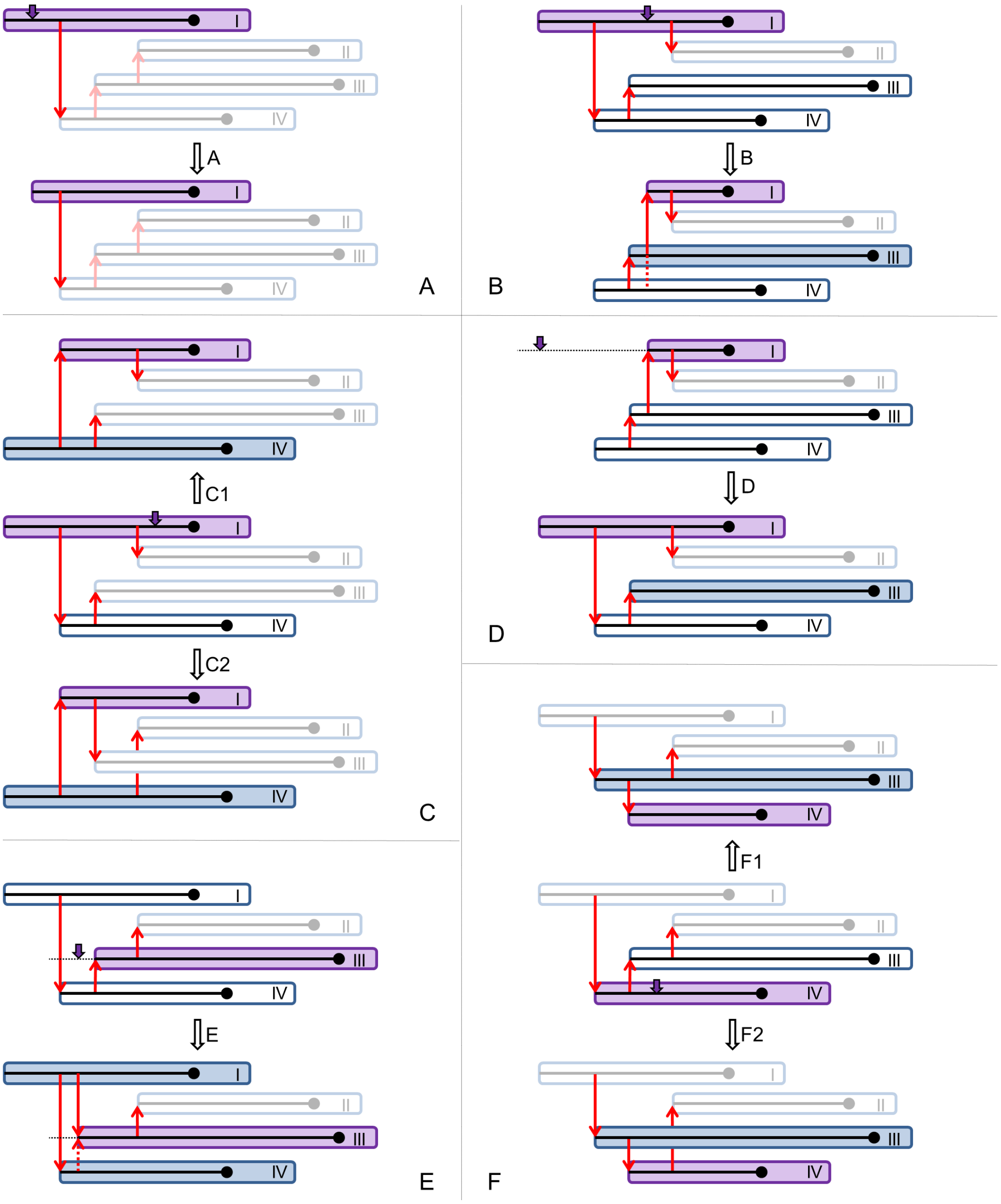

Fig M1. Graphics depicting proposal steps A-F for new transmission and phylogenetic trees. In panels A, B, D, and E, the initial situation is at the top, and the proposal below. In panels C and F, the initial situation is in the middle, and two alternative proposal above and below. Every panel shows an outbreak with four hosts, with red arrows indicating transmission: the purple host is the focal host, with the purple arrow indicating the proposal for the new infection time I_i'; filled hosts have a new phylogenetic mini-tree proposed; greyed-out hosts do not play a role in the proposal. (A) the focal host is the index case, and I_i' is before the first transmission event; (B) the focal host is the index case, and I_i' is after the first, but before the second secondary case; (C) the focal host is the index case and I_i' is after his second secondary case; (D) the focal host is not the index case and I_i' is before infection of the index case; (E) the focal host is not the index case and I_i' is before his first secondary case; (F) the focal host is not the index case and I_i' is after his first secondary case.

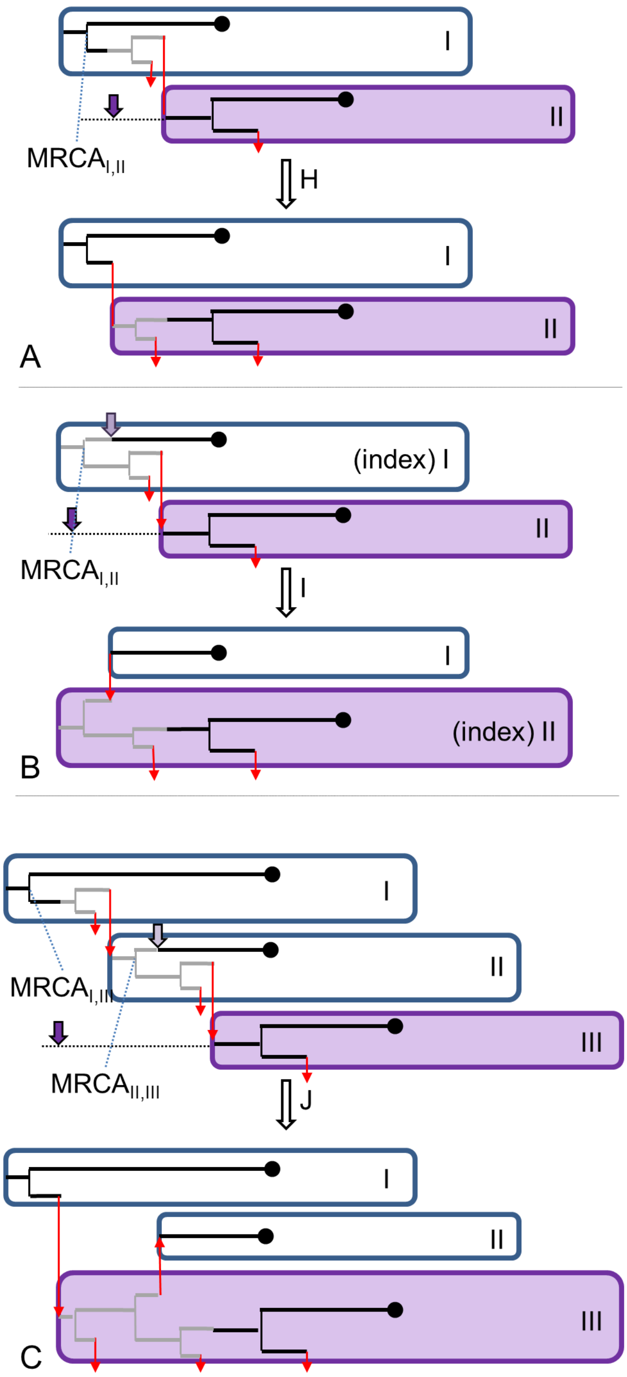

Fig M2. Graphics depicting proposal steps H-J for new transmission trees, keeping the phylogenetic tree unchanged. In all panels, the initial situation is at the top, and the proposal below. Every panel shows part of an outbreak, with red arrows indicating transmission to depicted or undepicted hosts. Only in panel B host I must be the index case. The purple host is the focal host, with the dark purple arrow indicating the proposal for the new infection time I_i'; the light purple arrow in panels B and C indicate the proposal for the new infection time I_j' of the focal host's infector. The grey parts of the phylogenetic tree are moved between the hosts. (H) the focal host is not the index case, and I_i' is after MRCA$_{I,II}$ of the focal host and his infector; (I) the focal host is not the index case, and I_i' is before MRCA$_{I,II}$ of the focal host and his infector (the index case), and I_j' is after MRCA$_{I,II}$; (J) the focal host is not the index case, and I_i' is before MRCA$_{II,III}$ of the focal host and his infector, but after the MRCA$_{I,III}$ of the focal host and his infector's infector; also, I_j' is after MRCA$_{II,III}$.
