# Supplementary figures and images for: A Bayesian inference method to estimate transmission trees with multiple introductions; applied to SARS-CoV-2 in Dutch mink farms
Source: PLoS Comput Biol. 2023 Nov 27;19(11):e1010928. doi: 10.1371/journal.pcbi.1010928 (PMC10703282; doi:10.1371/journal.pcbi.1010928)

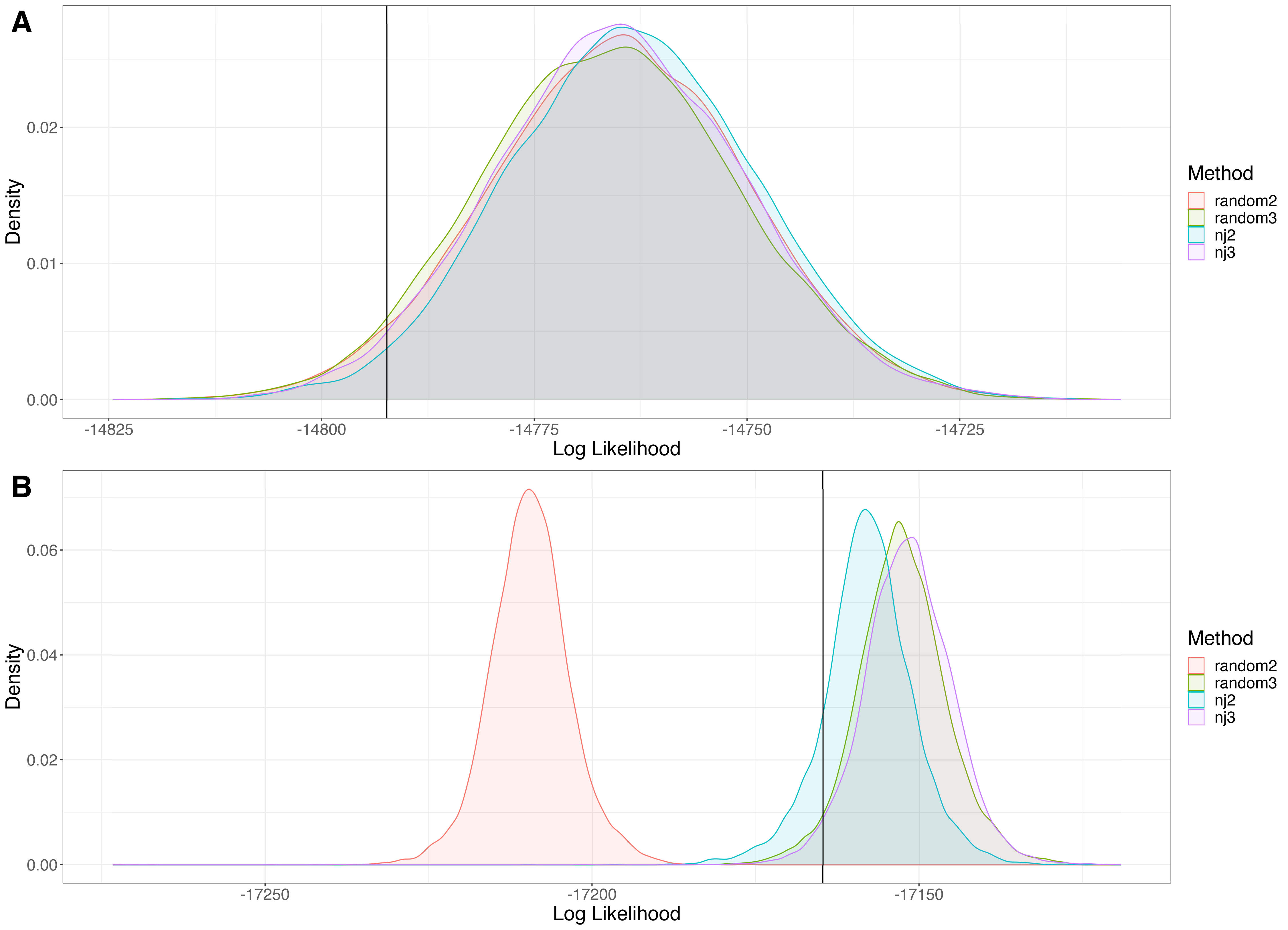

Supplement: S1 Fig — A: For low numbers of introductions (5 of the 20 hosts), there is no difference between methods in the posterior log-likelihood distribution. B: Higher numbers of introductions (15 of the 20 hosts), performance of MCMC with a random tree as initialization of the history host is inferior to either p(MC3), neighbour-joining tree initialization of the history host or the combination of both. Moreover, the simulated outbreak has a log-likelihood (the vertical black line) that is higher than the log-likelihood distribution of MCMC with a random tree as initialization. The latter gives the highest likelihood distribution and is chosen as default option in all analyses. ‘random’ is random tree initialization, ‘nj’ is neighbour-joining tree initialization, ‘2’ is MCMC and ‘3’ is p(MC3). The black lines are the log-likelihood values of the simulated outbreaks. (TIF) [file pcbi.1010928.s004.tif]

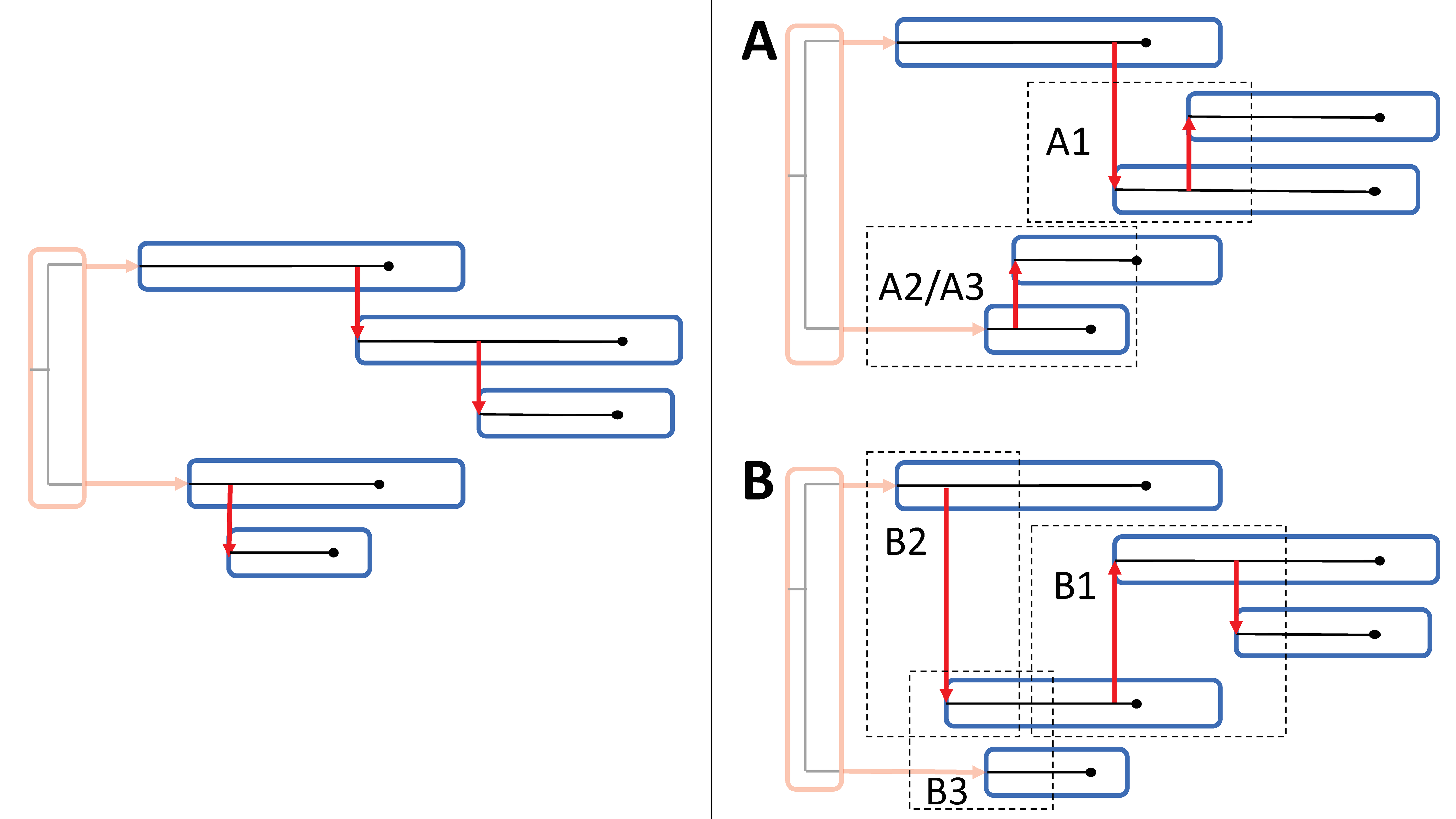

Supplement: S2 Fig — The left figure represents the transmission tree of a simulated outbreak with 5 cases; there are 2 introductions (clusters) and 3 transmission events. The right figure represents possible estimates of the transmission tree of the simulated outbreak. The vertical ordering of cases in the left and the right figures is identical. The upper right figure shows errors in which an incorrect infector is identified, but the incorrect infector belongs to the same cluster as the true infector (type A errors), the lower right figure represents incorrect identifications of the infector in which the incorrect infector belongs to a different cluster as the true infector (type B errors). In Type 1 errors neither the true infector nor the incorrect identified infector is an index case. For type 2 errors, the host is an index case in the simulated outbreak but not in the estimated outbreak. For type 3 errors, the host is not an index case in the simulated outbreak but is an index case in the estimated outbreak. (TIF) [file pcbi.1010928.s005.tif]

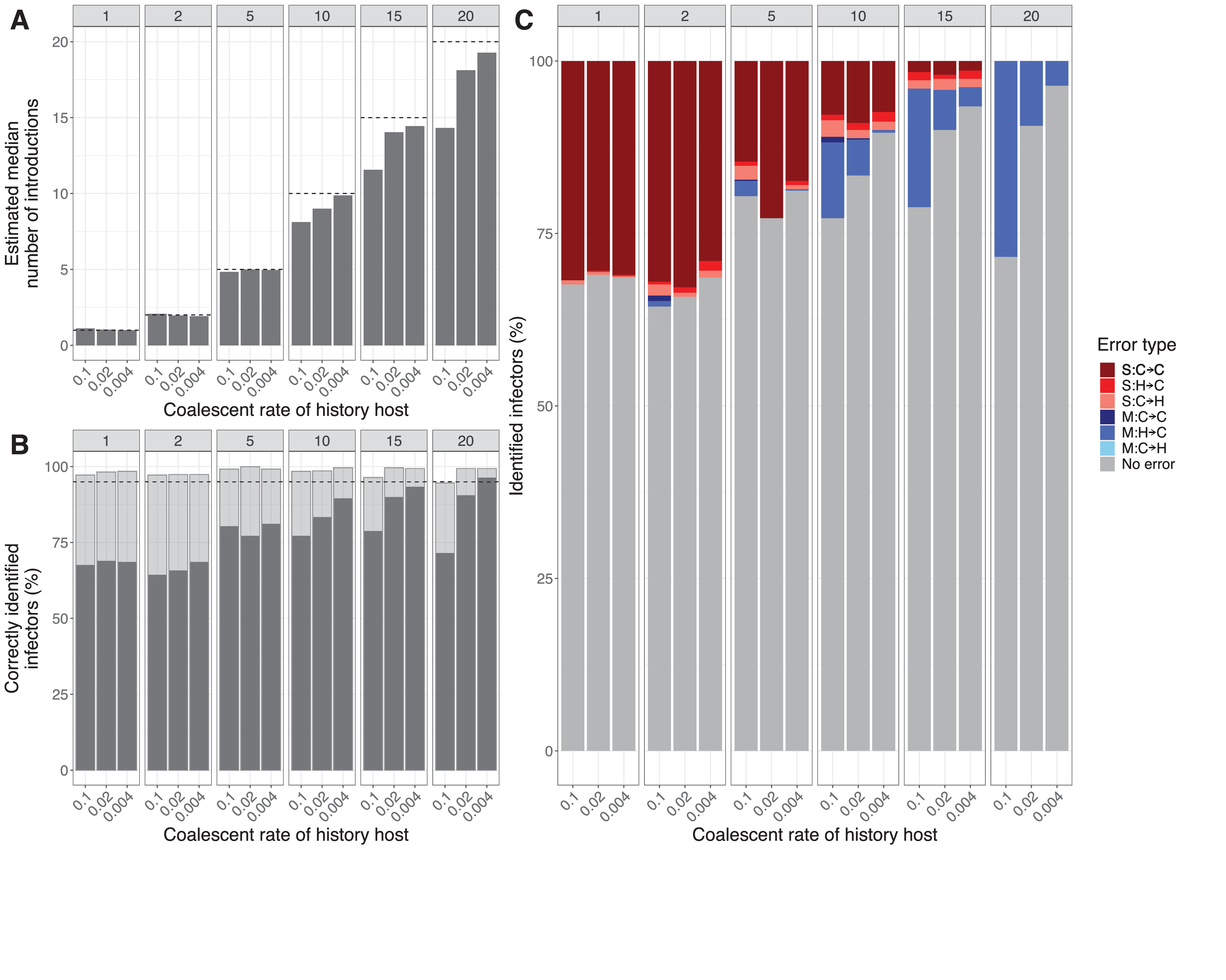

Supplement: S3 Fig — The model parameters are fixed at the simulation values. (A) The mean estimated median number of introductions. The black line indicates the simulated number of introductions. (B) Percentage of correctly identified infectors. The grey bar indicates cases for which the true infector has the highest posterior weight. The transparent bar indicates cases for which the true infector is contained in the smallest set of candidate infectors with at least 95% of the posterior weight. (C) Classification of the incorrectly identified infectors in the maximum credibility tree. The grey bars indicate the correctly identified infectors. S: single transmission cluster involved, M: multiple transmission clusters involved. C→C: simulated and inferred infectors are cases, H→C: simulated infector was history host, inferred infector is case, C→H: simulated infector was case, inferred infector is history host. (TIF) [file pcbi.1010928.s006.tif]

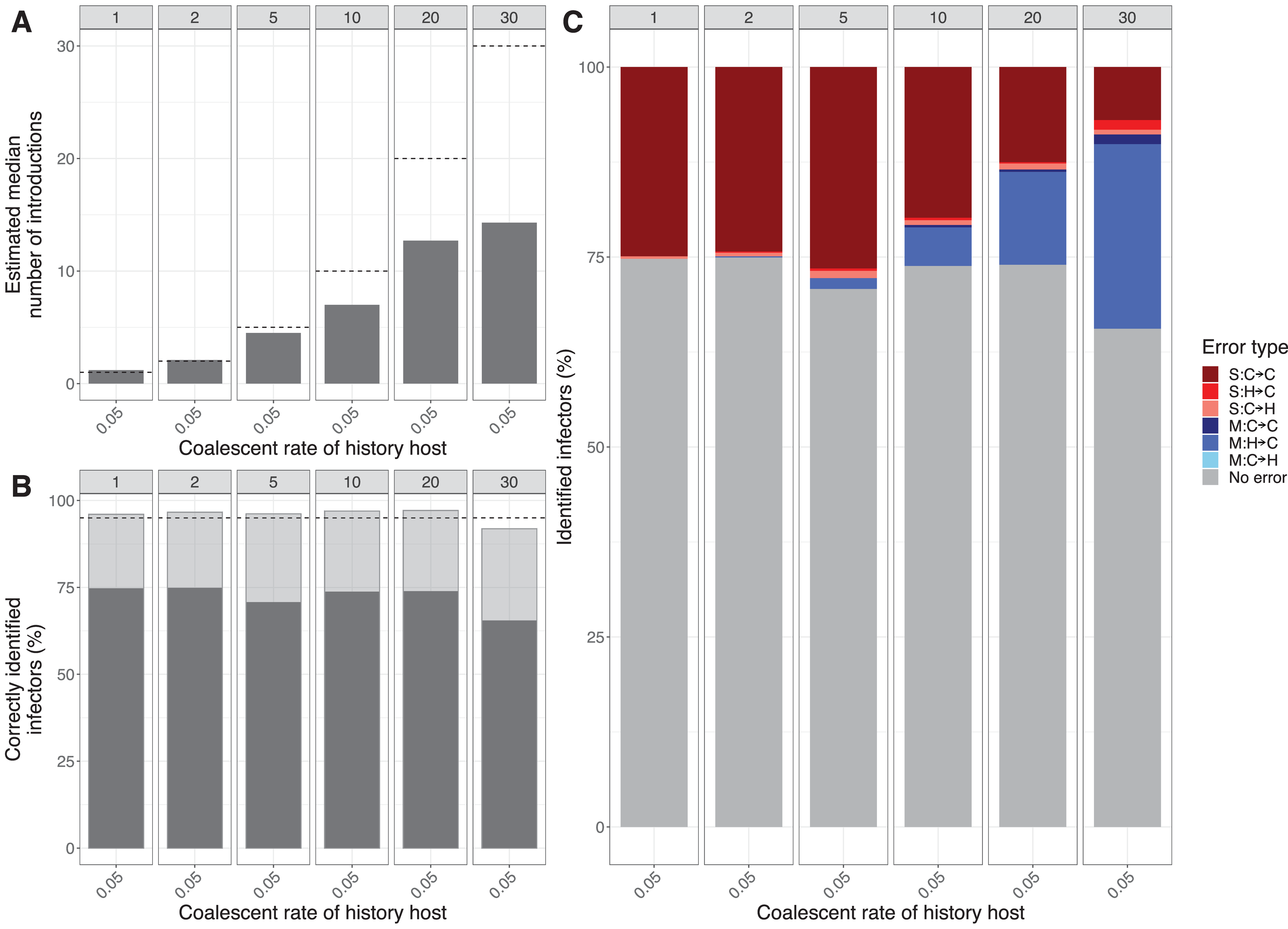

Supplement: S4 Fig — (A) The mean estimated median number of introductions. The black line indicates the simulated number of introductions. (B) Percentage of correctly identified infectors. The grey bar indicates cases for which the true infector has the highest posterior weight. The transparent bar indicates cases for which the true infector is contained in the smallest set of candidate infectors with at least 95% of the posterior weight. (C) Classification of the falsely identified infectors based on highest support. (C) Classification of the falsely identified infectors based on highest support. The grey bars indicate the correctly identified infectors. S: single transmission cluster involved, M: multiple transmission clusters involved. For the infector of a host: C→C: case becomes case, H→C: history becomes case, C→H: case becomes history. (TIF) [file pcbi.1010928.s007.tif]

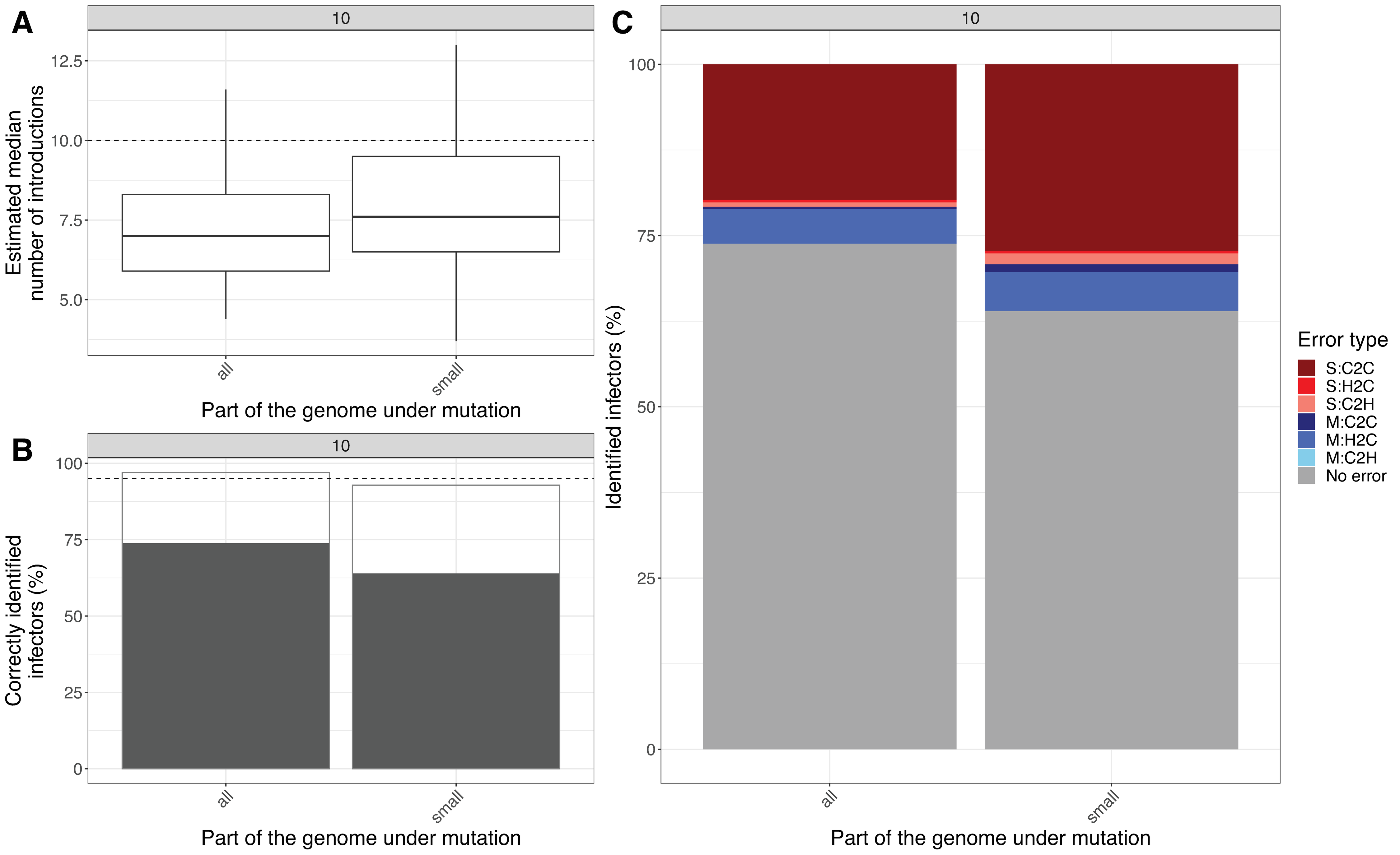

Supplement: S5 Fig — (A) The mean estimated median number of introductions. The black line indicates the simulated number of introductions. (B) Percentage of correctly identified infectors. The grey bar indicates cases for which the true infector has the highest posterior weight. The transparent bar indicates cases for which the true infector is contained in the smallest set of candidate infectors with at least 95% of the posterior weight. (C) Classification of the falsely identified infectors based on highest support. (C) Classification of the falsely identified infectors based on highest support. The grey bars indicate the correctly identified infectors. S: single transmission cluster involved, M: multiple transmission clusters involved. For the infector of a host: C→C: case becomes case, H→C: history becomes case, C→H: case becomes history. (TIF) [file pcbi.1010928.s008.tif]

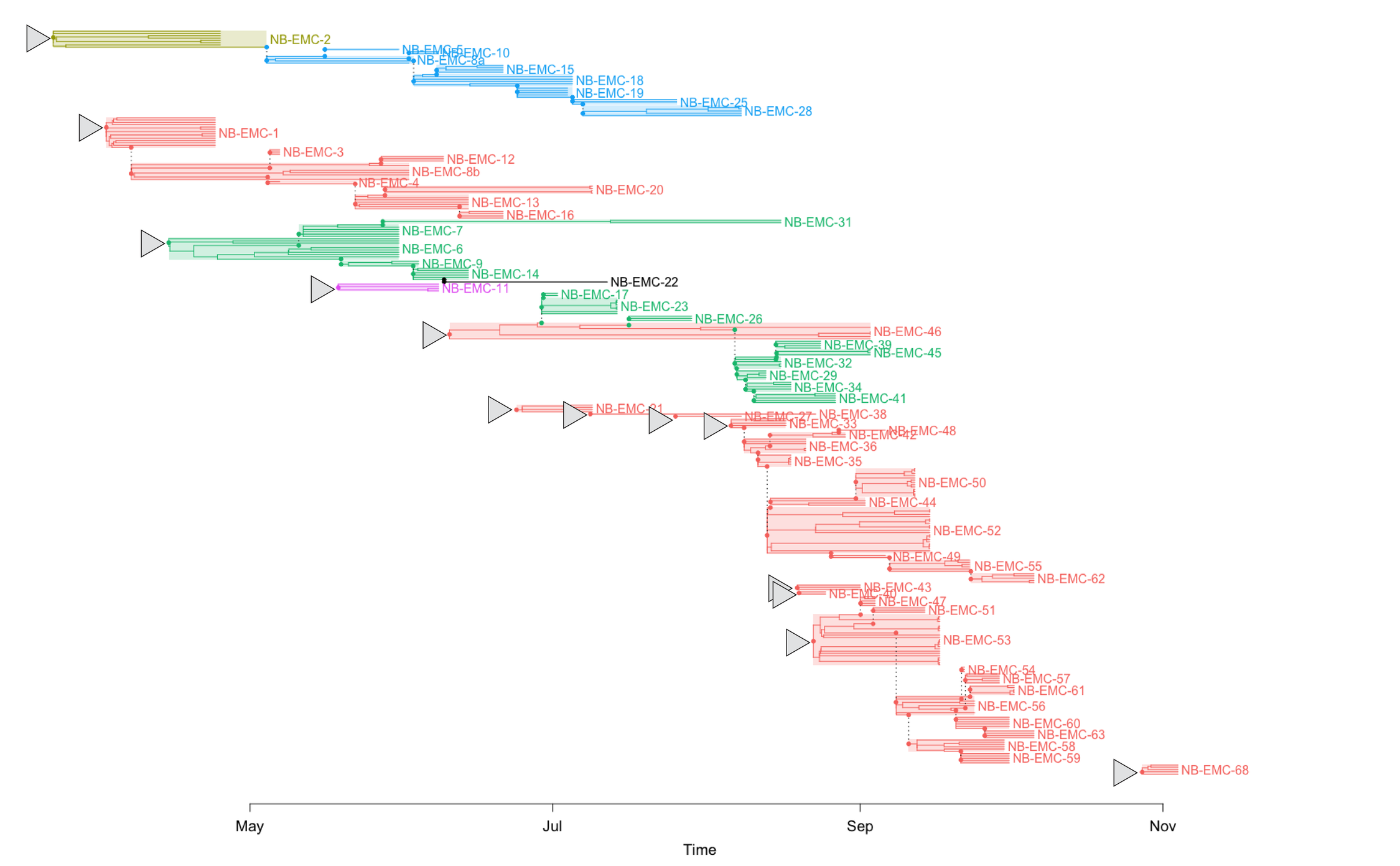

Supplement: S6 Fig — The farms are colored according to the clusters found by Lu et al. (2021): cluster A: red; cluster B; yellow, cluster C: green; cluster D: blue, cluster E: purple, cluster unknown: black. Cluster A is divided into 5 smaller clusters, with cluster A1 introduced in NB-EMC-1 and cluster A2 introduced in NB-EMC-46. (TIF) [file pcbi.1010928.s009.tif]

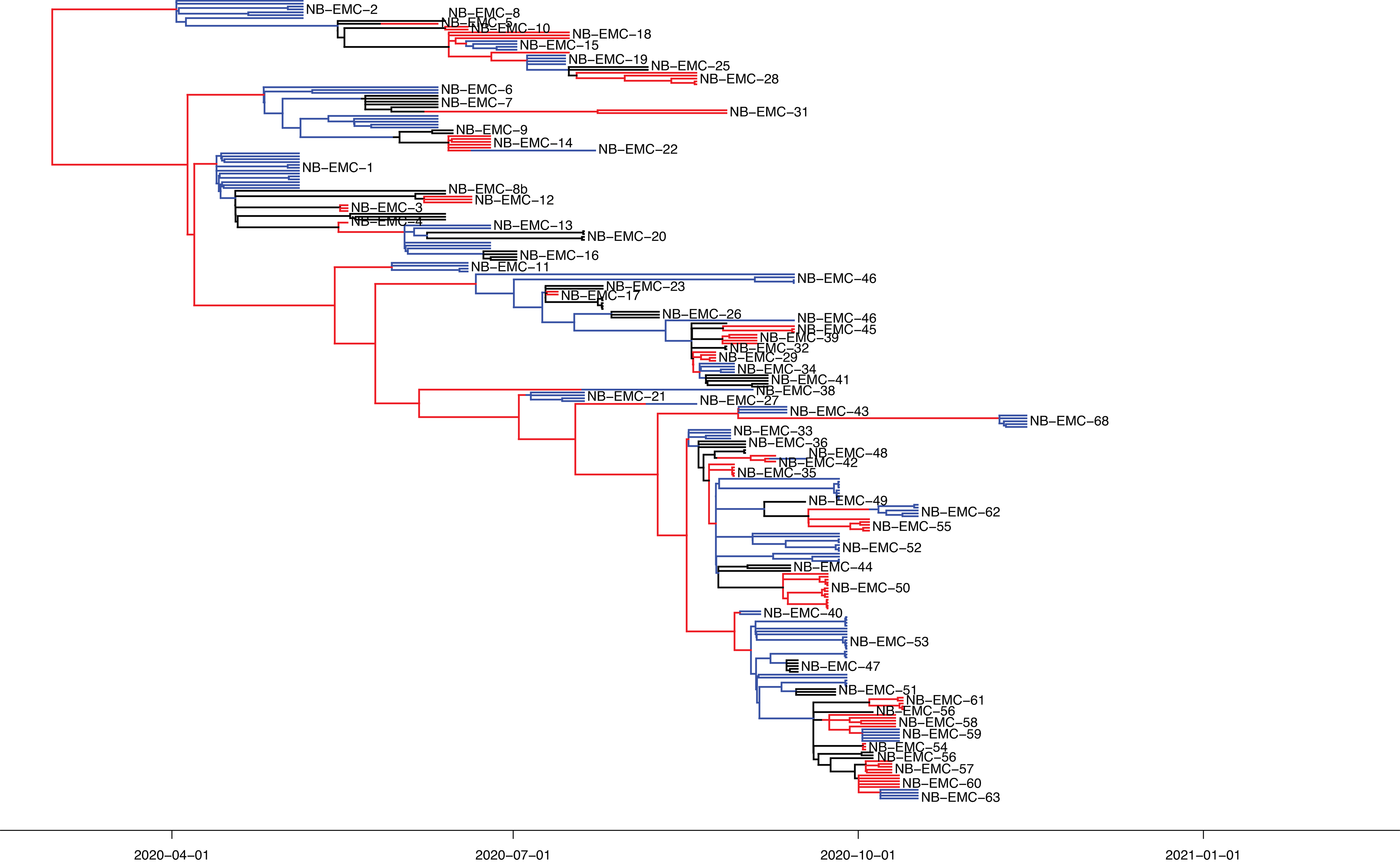

Supplement: S7 Fig — The history host is shown as the most-left red line, and the hosts are given in alternating colors. The black boxes represent the clusters in the transmission tree, with the lowest box the assumed bigger cluster with index case NB-EMC-46. (TIF) [file pcbi.1010928.s010.tif]

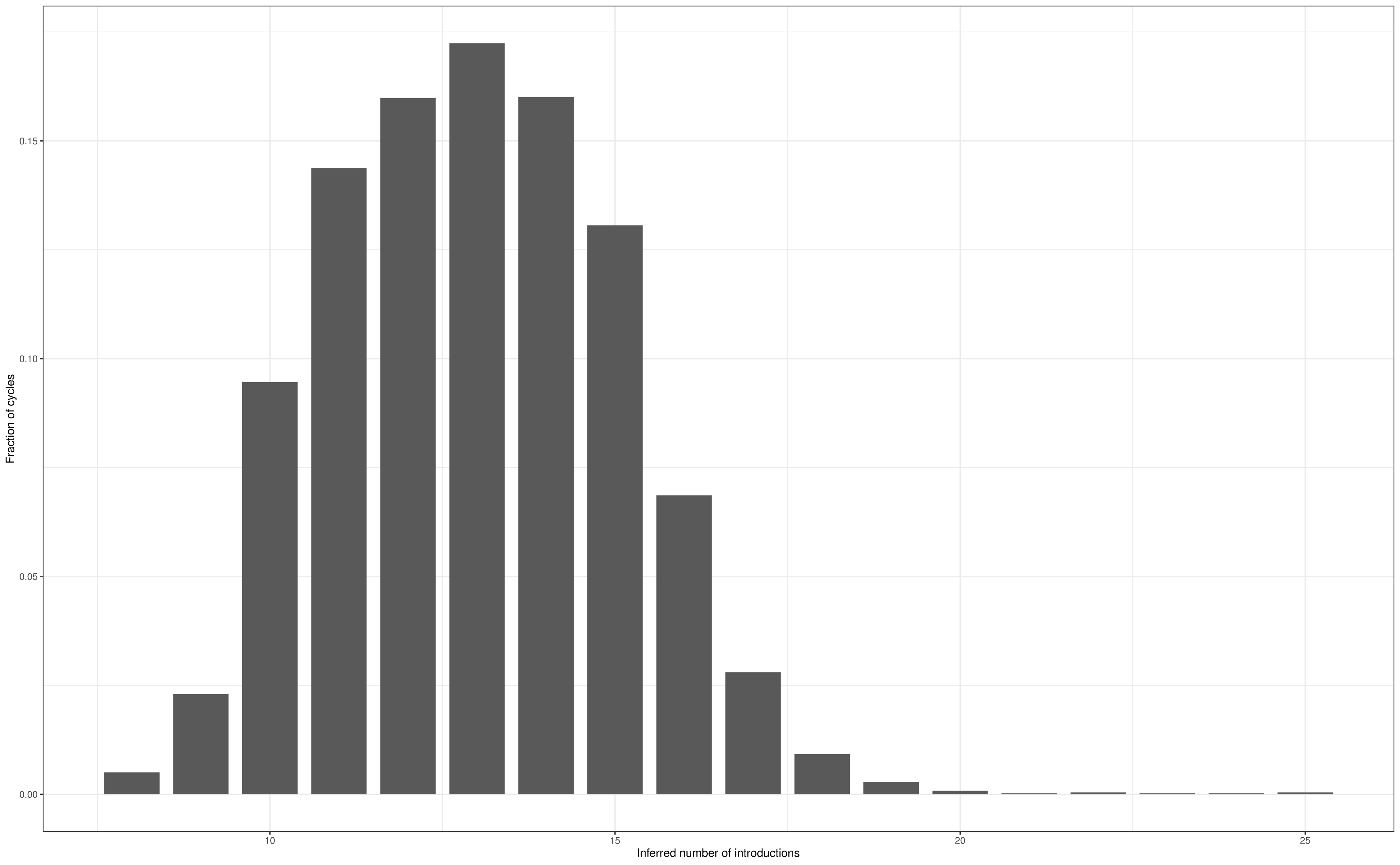

Supplement: S8 Fig — (TIF) [file pcbi.1010928.s011.tif]

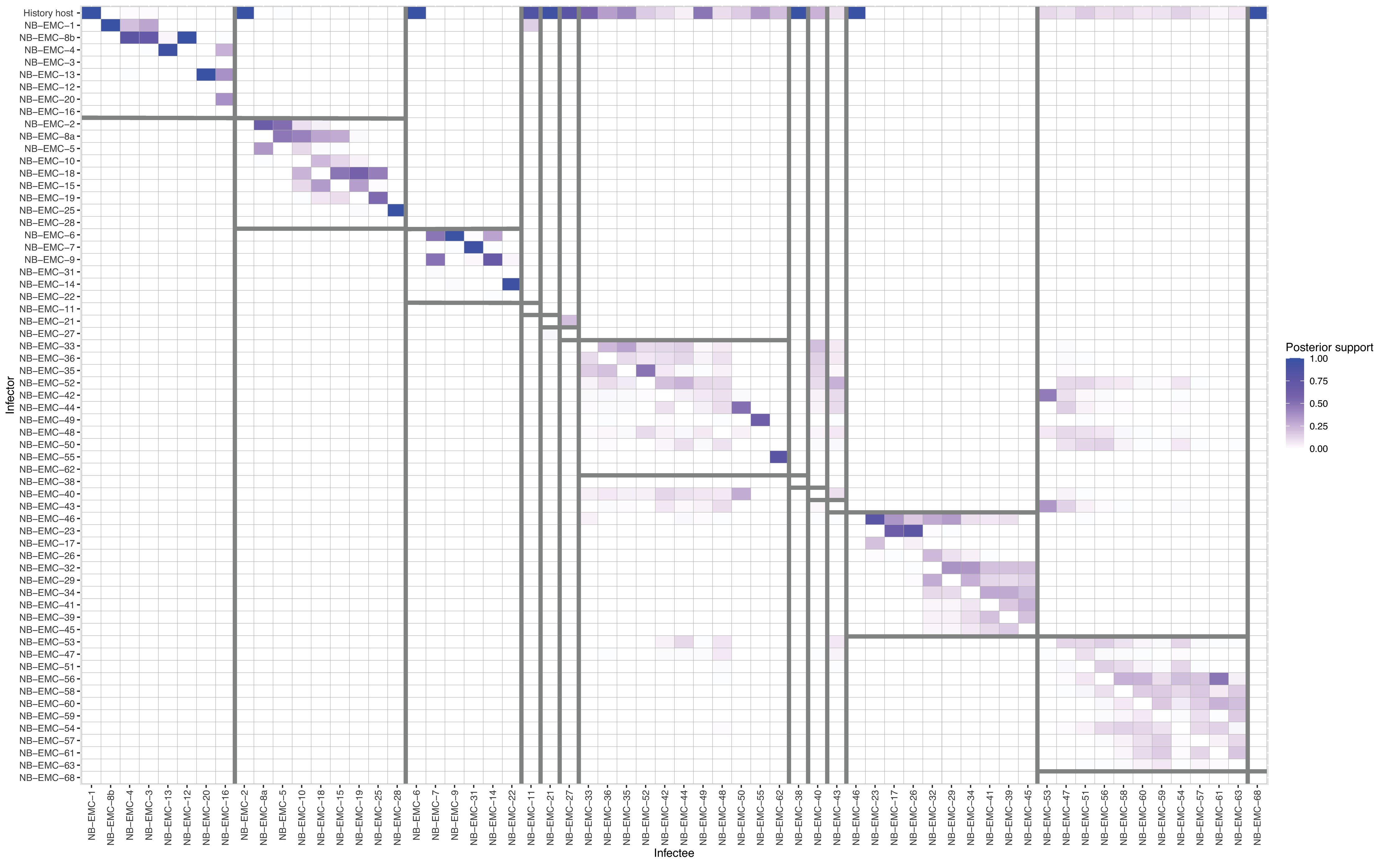

Supplement: S9 Fig — There is a high certainty of the index cases (infectees with the history host as infector) in the beginning of the outbreak. Transmission clusters with index cases NB-EMC-33 and NB-EMC-53 show more variation of the infectors, even outside their transmission cluster. Posterior support is shown from 0 (white) to 1 (blue). Hosts are ordered by transmission cluster and infection time. The grey bars show the transmission clusters. (TIF) [file pcbi.1010928.s012.tif]

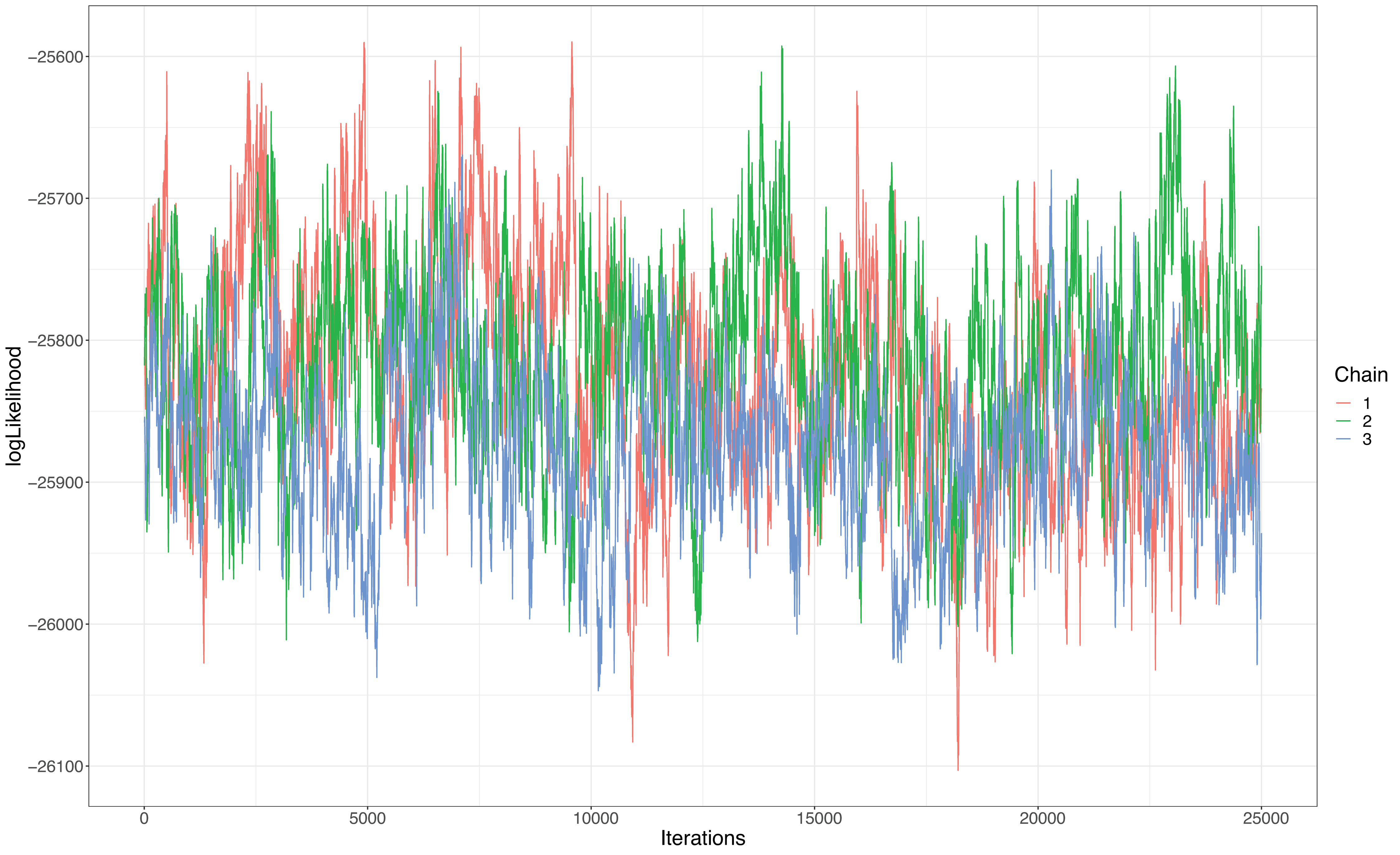

Supplement: S10 Fig — Traceplots are shown of the loglikelihood of 3 MCMC chains analyzing the SARS-CoV-2 outbreak in Dutch mink farms. (TIF) [file pcbi.1010928.s013.tif]
